# Increased sphingosine-1-phosphate improves muscle regeneration in acutely injured *mdx* mice

**DOI:** 10.1186/2044-5040-3-20

**Published:** 2013-08-01

**Authors:** Nicholas Ieronimakis, Mario Pantoja, Aislinn L Hays, Timothy L Dosey, Junlin Qi, Karin A Fischer, Andrew N Hoofnagle, Martin Sadilek, Jeffrey S Chamberlain, Hannele Ruohola-Baker, Morayma Reyes

**Affiliations:** 1 Department of Pathology, School of Medicine, University of Washington, Seattle, WA 98195, USA; 2 Department of Biochemistry, Institute for Stem Cell and Regenerative Medicine, University of Washington, Seattle, WA 98195, USA; 3 Department of Laboratory Medicine, School of Medicine, University of Washington, Seattle, WA 98195, USA; 4 Department of Chemistry, University of Washington, Seattle, WA 98195, USA; 5 Department of Neurology, Senator Paul D Wellstone Muscular Dystrophy Cooperative Research Center, University of Washington, Seattle, WA 98195, USA

## Abstract

**Background:**

Presently, there is no effective treatment for the lethal muscle wasting disease Duchenne muscular dystrophy (DMD). Here we show that increased sphingosine-1-phoshate (S1P) through direct injection or via the administration of the small molecule 2-acetyl-4(5)-tetrahydroxybutyl imidazole (THI), an S1P lyase inhibitor, has beneficial effects in acutely injured dystrophic muscles of *mdx* mice.

**Methods:**

We treated *mdx* mice with and without acute injury and characterized the histopathological and functional effects of increasing S1P levels. We also tested exogenous and direct administration of S1P on *mdx* muscles to examine the molecular pathways under which S1P promotes regeneration in dystrophic muscles.

**Results:**

Short-term treatment with THI significantly increased muscle fiber size and extensor digitorum longus (EDL) muscle specific force in acutely injured *mdx* limb muscles. In addition, the accumulation of fibrosis and fat deposition, hallmarks of DMD pathology and impaired muscle regeneration, were lower in the injured muscles of THI-treated *mdx* mice. Furthermore, increased muscle force was observed in uninjured EDL muscles with a longer-term treatment of THI. Such regenerative effects were linked to the response of myogenic cells, since intramuscular injection of S1P increased the number of *Myf5*^*nlacz/+*^ positive myogenic cells and newly regenerated myofibers in injured *mdx* muscles. Intramuscular injection of biotinylated-S1P localized to muscle fibers, including newly regenerated fibers, which also stained positive for S1P receptor 1 (S1PR1). Importantly, plasma membrane and perinuclear localization of phosphorylated S1PR1 was observed in regenerating muscle fibers of *mdx* muscles. Intramuscular increases of S1P levels, S1PR1 and phosphorylated ribosomal protein S6 (P-rpS6), and elevated EDL muscle specific force, suggest S1P promoted the upregulation of anabolic pathways that mediate skeletal muscle mass and function.

**Conclusions:**

These data show that S1P is beneficial for muscle regeneration and functional gain in dystrophic mice, and that THI, or other pharmacological agents that raise S1P levels systemically, may be developed into an effective treatment for improving muscle function and reducing the pathology of DMD.

## Background

Duchenne muscular dystrophy (DMD) is a muscle wasting disease for which there is no cure. This severe X-linked recessive disease affects 1 in 3,500 male births [1]. In dystrophic muscles, rounds of contractions result in degeneration/regeneration cycles. In turn, dystrophic muscle cannot regenerate sufficiently to overcome degeneration, leading to muscle wasting over time. Since no effective treatment presently exists and the immune response to dystrophin has hampered gene therapy approaches, new advances for the treatment of DMD are imperative [2,3].

Previously, sphingosine-1-phosphate (S1P) has been implicated in muscle repair, satellite cell proliferation, myoblast differentiation *in vitro* and in non-diseased mouse models *in vivo*[2,4-6]. These essential roles for S1P in skeletal muscle regeneration suggested that elevation of S1P may have therapeutically beneficial effects in models of disease [7]. More recently, S1P has been shown beneficial for activating satellite cells in dystrophic muscles [8]. Furthermore, an unbiased genetic modifier screen in *Drosophila* revealed that by increasing S1P levels via reduction of the lipid phosphate phosphatase 3 (LPP3) homolog, wunen, or the S1P lyase, sply, prevents to a large degree dystrophic muscle wasting in flies [9]. In mice, elevation of S1P by the genetic reduction of S1P lyase can be phenocopied pharmacologically via treatment with the small molecule 2-acetyl-4(5)-tetrahydroxybutyl imidazole (THI) [10,11]. Furthermore, in *Drosophila,* THI treatment also significantly suppresses the dystrophic muscle phenotype [9].

Utilizing the *mdx* mouse model, we initiated studies on the effect of increasing S1P levels in dystrophic mice, and found that short-term treatment with THI improves muscle integrity and function following acute injury with cardiotoxin (CTX). THI treatment also leads to significant improvements of the pathology of dystrophic muscles, as indicated by the reduced accumulation of fibrosis and fat deposition in acutely injured muscles. In turn, intramuscular injection of S1P resulted in an increased number of myogenic cells and newly regenerating fibers *in vivo*. S1P receptor 1 (S1PR1) is expressed by many muscle cell types, particularly muscle fibers, and phosphorylated S1PR1 is localized in the plasma membrane and intracellularly (perinuclear localization) of muscle fibers. Intramuscular S1P administration results in increased levels of total and phosphorylated S1PR1 and ribosomal protein S6 (rpS6). This suggests that increases in fiber size are mediated by anabolic pathways that promote greater skeletal muscle mass and function, potentially through S1PR1 signaling. Furthermore, *ex vivo* administration of S1P improved specific force in uninjured dystrophic muscle. Similarly, longer-term THI treatment of uninjured young *mdx* mice resulted in increased extensor digitorum longus (EDL) muscle force in the absence of CTX injury. Altogether, S1P acts at multiple levels in muscles, particularly in myogenic cells and muscle fibers, and collectively the actions of S1P in muscle are beneficial for regeneration in the setting of muscular dystrophy.

## Methods

### Animal procedure

Experiments involving animals were undertaken in accordance with approved guidelines and ethical approval from the Institutional Animal Care and Use Committee, University of Washington, Seattle, WA, USA.

### THI injections in injured mice

**Figure 1:**
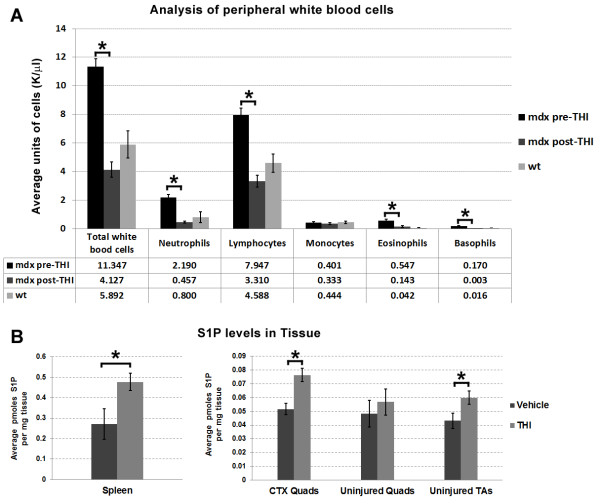
**IP injection of THI reduces peripheral blood leukocytes and increases S1P levels in most tissues. (A)** Leukocytes were analyzed from the peripheral blood of 1.5-MO *mdx*^*4cv*^ mice (n = 3) before and 12 hours following treatment with THI (2 × 250 μl 0.15 mg/ml IP injections, 6 hours apart). IP administration of THI significantly reduced circulating leukocytes to values below or near age-matched wt (n = 4). The average value of each population is listed in the table below the bar graph. Values between pre and post THI, and wt were also significant by ANOVA (*P* <0.05) for all leukocytes except monocytes. **(B)***mdx*^*4cv*^ mice (n = 6, 5-MO) were treated with THI or vehicle for 3 days (2 × 250 μl 0.15 mg/ml IP injections per day) following CTX injury to assess changes in S1P muscle content. Muscles and spleens were harvested on day 4 post injury for S1P analysis by LC-MS/MS. Results indicate S1P levels in spleen and injured quadriceps (quads) were significantly elevated with THI treatment. Interestingly, uninjured quadriceps did not show a significant increase of S1P, whereas uninjured TA muscles did. *P <0.05 by student’s *t*-test. Error bars represent SEM. CTX, cardiotoxin; IP, intraperitoneal; LC-MS/MS, liquid chromatography-tandem mass spectrometry; MO, month-old; S1P, sphingosine-1-phoshate; SEM, standard error of the mean; TA, tibialis anterior; THI, 2-acetyl-4(5)-tetrahydroxybutyl imidazole; wt, wild type.

**Figure 2:**
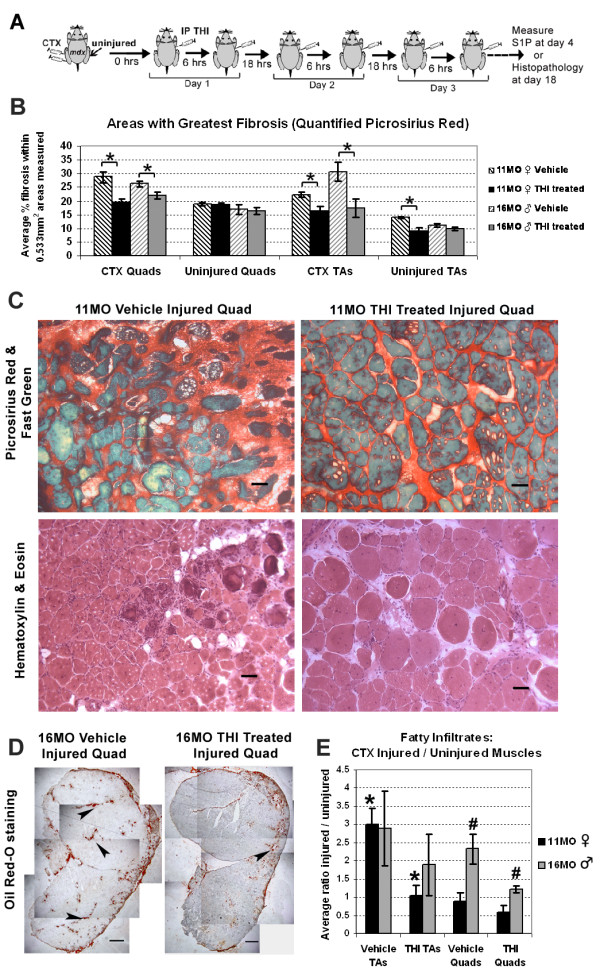
**Dystrophic pathology following muscle injury is improved with THI treatment. (A)** Experimental schematic of THI (0.075 μg/day) and PBS (vehicle)-treated *mdx* mice injected IP twice daily for the first 72 hours following CTX injury. Muscles from aged *mdx*^*4cv*^ mice (n = 7, THI-treated: 3 × 11-MO females, 4 × 16-MO males; n = 6 vehicle-treated: 3 × 11-MO females, 3 × 16-MO males) were harvested for histopathology analysis 18 days post CTX injury. **(B)** Histological quantification of picrosirius red staining indicates lower fibrotic accumulation following injury in both TA and quadriceps (quads) muscles from mice treated with THI. For CTX-injected muscles, damaged regions of muscle (for example fields with the greatest accumulation of sirius red staining) were quantified for both THI and vehicle-treated mice. The level of fibrosis was not significantly different between treated and control (vehicle) uninjured quadriceps; however, uninjured TA muscles from 11-MO THI-treated mice had lower fibrosis compared to control TA muscles. For each muscle, three separate sections (200 μm apart in longitudinal distance) were analyzed. **(C)** Representative photographs of injured quadriceps stained with picrosirius red and fast green depict collagen deposition (red staining), while muscle morphology and organization is depicted with hematoxylin and eosin staining. Scale bars = 50 μm. **(D)** Oil Red O staining depicts fat deposits (arrows) over the entire CSA of THI-treated and vehicle-injured quadriceps from 16-MO males. Scale bars = 500 μm. **(E)** The ratio of fat deposition in injured TAs over uninjured contralateral TAs quantified from Oil Red O staining was significantly reduced in THI-treated versus control animals in 11-MO (*) but not 16-MO *mdx*^*4cv*^ mice. In contrast, the ratio of injured over uninjured fat deposits in quadriceps was significantly reduced in 16-MO (#) but not in 11-MO *mdx* mice. *P <0.05, **P <0.01 by student’s *t*-test. Error bars represent SEM. CSA, cross-sectional area; CTX, cardiotoxin; IP, intraperitoneal; MO, month-old; SEM, standard error of the mean; TA, tibialis anterior; THI, 2-acetyl-4(5)-tetrahydroxybutyl imidazole.

**Figure 3:**
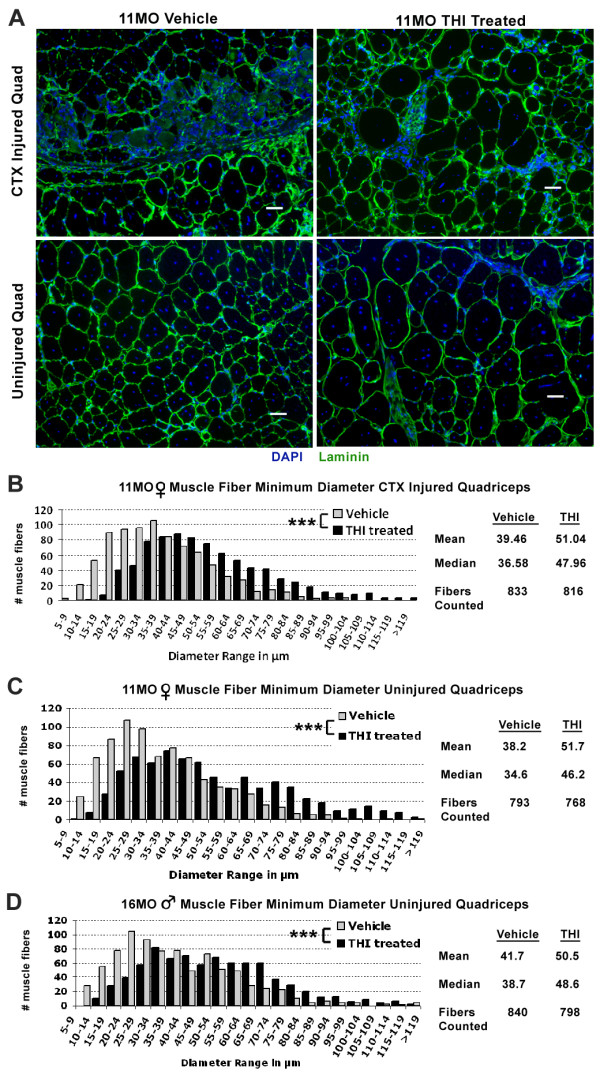
**Elevating S1P levels with THI increases muscle fiber size. (A)** Staining for laminin (green) and DAPI (blue) depict a dramatic increase in muscle fiber size in both injured and uninjured quadriceps (quads) with THI treatment. Depicted are quadriceps muscles from 11-MO *mdx*^*4cv*^ mice. Scale bars = 50 μm. **(B,C,D)** Quantification of minimum muscle fiber diameter reveals a significant increase in myofiber size in THI-treated animals. Increased myofiber diameter was observed in both **(B)** injured and **(C)** uninjured quadriceps from THI-treated 11-MO *mdx*^*4cv*^ mice, whereas only **(D)** uninjured quadriceps in THI-treated 16-MO *mdx*^*4cv*^ mice showed increased myofiber size compared to vehicle controls. As indicated by the distributions, mean and median values of muscle fiber minimum diameters, there is an overall increase in muscle fiber size with THI treatment. Quantifications were undertaken in random fields in both injured and uninjured muscles in order to obtain an overall representation of fiber size increase for each muscle.*P <0.05, ***P <0.0005 by student’s *t*-test. Error bars represent SEM. DAPI, 4',6-diamidino-2-phenylindole; MO, month-old; S1P, sphingosine-1-phoshate; SEM, standard error of the mean; THI, 2-acetyl-4(5)-tetrahydroxybutyl imidazole.

**Figure 4:**
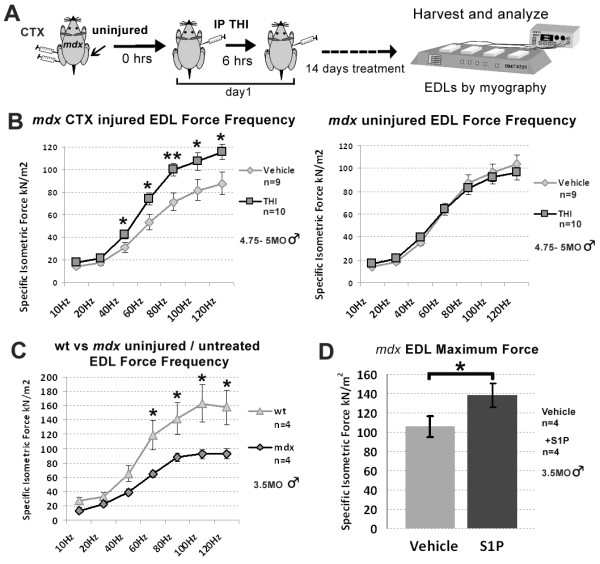
**S1P promotes functional improvement of *mdx *(*C57BL/10ScSn-Dmd***^***mdx/J***^**) muscle. (A)** Experimental schematic of longer-term, 14-day treatment of THI or PBS (vehicle) following CTX injury. THI was administered following the aforementioned dose and injection regimen. Following treatment, EDL muscles were harvested and specific isometric force was analyzed by *in vitro* myography from both injured and uninjured limbs. **(B)** Force frequency analysis reveals that EDL muscles isolated from injured limbs of THI-treated animals (n = 10) have significantly greater specific force compared to injured vehicle controls (n = 9). **(C)** Analysis of untreated and uninjured wt (*C57BL/10ScSn*) and *mdx* (*C57BL/10ScSn-Dmd*^*mdx/J*^) indicate specific force improved in injured but not uninjured THI-treated EDL muscles. **(D)** Incubation of uninjured and untreated *mdx* (*C57BL/10ScSn-Dmd*^*mdx/J*^) EDL muscles with a high concentration of S1P (10 μM) leads to a significant increase in maximal specific force. *P <0.05, **P <0.005 by student’s *t*-test. Error bars represent SEM. CTX, cardiotoxin; EDL, extensor digitorum longus; S1P, sphingosine-1-phoshate; SEM, standard error of the mean; THI, 2-acetyl-4(5)-tetrahydroxybutyl imidazole; wt, wild type.

Peripheral blood cells from 1.5-month-old (MO) wild type (wt) *C57BL/k6* and *mdx* mice on a *C57BL/k6* background (*B6Ros.Cg-Dmd*^*mdx-4Cv*^*/J*, herein referred to as *mdx*^*4cv*^) were analyzed (Figure 1A). Blood was collected before and 12 hours following the last of two 250 μl intraperitoneal (IP) injections of 0.15 mg/ml THI in PBS. Injections were 6 hours apart. This injection regimen and dose was repeated for all subsequent experiments involving THI, but for longer-treatment durations as outlined. Six 5-MO *mdx*^*4cv*^ males were used for the experiments in Figure 1B, and Additional file 1: Figure S1 and S2. For Figures 2 and 3, and Additional file 1: Figures S3 to S7, six 11-MO females and seven 16-MO males *mdx*^*4cv*^ were used for these experiments. In these mice, the left tibialis anterior (TA) and quadriceps femoris (quads) were injured with 10 nM CTX (Calbiochem, Darmstadt, Germany) from *Naja nigricollis*. Once more, THI-treated mice were injected IP with 250 μl 0.15 mg/ml THI in PBS, twice daily (injections 6 hours apart) immediately after injury and for the first 3 days following injury. The vehicle controls were injected IP with PBS. On day 4 post injury, 5-MO *mdx*^*4cv*^ animals were euthanized for S1P and creatine kinase (CK) analysis. On day 17 post CTX, 11-MO and 16-MO *mdx*^*4cv*^ mice were also injected IP with 1% Evans Blue dye (EBD) to label persistently damaged (dye permeable) muscle fibers [12], and euthanized on day 18 post injury for histopathology analysis. Muscles for S1P and expression analysis (from 5-MO *mdx*^*4cv*^) were frozen directly in liquid nitrogen, while muscles taken for histopathology were frozen under liquid nitrogen cooled isopentane in optimal cutting temperature (OCT) compound. All myofibers were measured for the minimum diameters on the cross-sections of mouse quadriceps muscle using ImageJ software (Bethesda, MD, USA). Between 750 and 850 myofibers were counted for three mice treated with PBS or THI, with or without CTX injury. For functional analysis outlined in Figure 4B, 4.75- to 5-MO male *mdx* on a C57BL/10 background (*C57BL/10ScSn-Dmd*^*mdx/J*^) were used for the 14-day treatment of THI or vehicle. Following the same dose and treatment regimen, *mdx* were treated with THI (n = 10) or vehicle (n = 9) for 14 days following CTX injury to left TAs and quadriceps. The same *mdx* strain was compared to wt *C57BL/10* animals in Figure 4C and for exogenous S1P treatment depicted in Figure 4D. Animals used to evaluate the degree of CTX injury in EDL (Additional file 1: Figure S8) were 4-MO female *mdx* (n = 4, *C57BL/10ScSn-Dmd*^*mdx/J*^ background), injected in left TAs with CTX and with approximately 3 μl India ink, added to the tip of the needle to mark injection penetration. Following CTX injections, mice were immediately injected IP with 1% EBD. Both left (injured) and contralateral uninjured TA and EDL muscles were harvested and frozen in OCT compound 12 hours post injury.

### THI treatment in drinking water of young, uninjured *mdx* mice

Beginning at 4 weeks of age, male *mdx*^*4cv*^ were treated with THI (n = 4) or vehicle (n = 3) for 4 weeks, and analyzed by EDL myography at 8 weeks of age. For this treatment we followed the dose and conditions described by Schwab *et al*. [11]. Briefly, 50 mg/l THI was administered *ad libitum*. The vehicle consisted of water at pH 2.8 containing 10 g/l glucose.

### Peripheral blood cell analysis

Blood was collected via retro-orbital blood collection using heparinized capillaries and transferred to blood collection tubes containing a final concentration of 1.6 mg/ml EDTA (SARSTEDT, Nümbrecht, Germany) for analysis. Analysis of whole blood was undertaken with 20 μl per sample using the Hemavet 950 FS system (Drew Scientific, Dallas, TX, USA).

### Analysis of gene expression by quantitative reverse transcription-PCR (RT-PCR)

Total RNA (RNeasy Kit, Qiagen, Venlo, Netherlands) was prepared from *mdx*^*4cv*^ TA muscles homogenized under liquid nitrogen by mortar and pestle. Methods for RNA isolation and cDNA generation were in accordance with manufacturer’s protocols using reverse transcriptase (Applied Biosystems, Carlsbad, CA, USA) as previously described [13]. RNA (0.5 μg) was reverse transcribed using the Omniscript RT Kit (Qiagen). For reverse transcription-PCR (RT-PCR), 10 ng cDNA was combined with SYBR Green (Thermo Scientific, Waltham, MA, USA) following published conditions and primer sequences for S1P-related genes by Grabski *et al*. [14] and by Au *et al*. [15] for *18S*.

### Functional analysis: myography

Animals treated with THI or PBS (vehicle) via IP injection as aforementioned for 14 days were analyzed between 1 and 4 days following the final day of injection. Prior to euthanasia animals were anesthetized with 0.5 mg/g weight avertin diluted in PBS. EDLs were then excised and equilibrated in Ringer’s solution (120 mM NaCl, 4.7 mM KCl, 3.15 mM MgCl_2_, 1.3 mM NaH_2_PO_4_, 25 mM NaHCO_3_, 11 mM glucose, 1.25 mM CaCl_2_, pH 7.2) with 95% O_2_/5% CO_2_ for a minimum of 15 minutes prior to stimulation [16]. For assessment of direct S1P administration, EDL muscles from uninjured and untreated 3.5-MO male *mdx* (*C57BL/10ScSn-Dmd*^*mdx/J*^) were incubated with oxygenated Ringer’s solution containing 10 μM S1P or vehicle (PBS with 4 mg/ml fatty acid free BSA) for 15 minutes prior to stimulation [16]. All functional experiments were carried out with buffer solutions at 25°C under constant oxygenation. Myography was conducted using a 820S myograph (DMT, Ann Arbor, MI, USA) and data was recorded using a PowerLab 4/30 acquisition system with LabChart Pro software v7.3.1 (both from ADInstruments, Dunedin, New Zealand). Stimulations were conducted with S88X dual systems (Grass Technologies, Middleton, WI, USA). Muscles were stimulated to establish optimal fiber length (Lf) and voltage at which maximum tetanic force was measured at 120 Hz using 4.15 ms pulses within 450 ms train duration [17]. Force frequency was carried out using the same pulse duration at 10, 20, 40, 60, 80, 100 and 120 Hz, as outlined in the x-axis of Figure 3B. Specific force was calculated as previously described [18] by normalizing to the muscle cross-sectional area (CSA). CSA is the quotient of dry muscle mass (mg) over Lo (mm), which is defined as the product of Lf with the fiber length ratio (0.44 for EDL) and mammalian muscle density (1.06 mg/mm^3^).

### Measurement of S1P in mouse tissue

S1P was quantified in tissue after homogenization and extraction using liquid chromatography-tandem mass spectrometry (LC-MS/MS). Tissue was pulverized in liquid nitrogen using a mortar and pestle. Collected tissue was weighed and an internal standard (C17 base D-erythro-sphingosine-1-phosphate in methanol (Avanti Polar Lipids, Alabaster, AL, USA)) was added at 1 pmol/mg tissue. Tissue was then vortexed/extracted in 16 volumes (mg/μl) of acetonitrile:water (80:20, v/v) for 10 minutes at room temperature. Supernatants were collected after centrifugation (10 minutes at 14,000 rpm) and concentrated to dryness using a SpeedVac Concentrator (Thermo Scientific). Pellets were resuspended in methanol to a calculated concentration of 0.05 μM C17 base D-erythro-sphingosine-1-phosphate. Then 10 μl was analyzed by LC-MS/MS using C17 base D-erythro-sphingosine-1-phosphate plus C18 base D-erythro-sphingosine-1-phosphate (both at 0.05 μM) as a standard. Separation of analytes was undertaken by liquid chromatography using a Chromolith RP-C18e 100 × 2 mm column (EMD, Gibbstown, NJ, USA) and analysis by tandem mass spectrometry with a Quattro Micro mass spectrometer (Waters, Milford, MA, USA) in positive ion mode. The HPLC gradient using two pumps was linear from 50% MeOH to 99% MeOH using solvent A (water, 0.1% formic acid) and solvent B (MeOH, 0.1% formic acid) over 1 minute at a flow rate of 0.35 ml/min. To wash the column, the gradient was repeated twice before equilibrating for 3 minutes before running the next sample. The transitions analyzed were 380.25 >264.50 and 380.25 >82.00 for endogenous S1P, and 366.25 >250.50 and 366.25 >82.00 for internal standard with a dwell time of 0.07 seconds. Data collection was by MassLynx software (Waters) and processed with QuanLynx software (Waters).

### Measurement of S1P in mouse plasma

S1P was quantified in plasma using butanol extraction and liquid LC-MS/MS [19]. Internal standard (5 μl 3 μM C17 base D-erythro-sphingosine-1-phosphate in ethanol (Avanti Polar Lipids)) was added to 10 μl EDTA-anticoagulated plasma and mixed thoroughly on an orbital shaker (Thermomixer, Eppendorf, Hauppauge, NY, USA) for 10 minutes at 1,400 rpm at 20°C. The sample was then acidified using 50 μl 30 mM citric acid/40 mM Na_2_HPO_4_, pH 4.0, and extracted for 10 minutes at 1,400 rpm at 20°C with 125 μl water-saturated butanol (Fisher Scientific, Waltham, MA, USA). The butanol layer was removed and lyophilized in a centrifugal evaporator at 20°C. The residue was stored at −20°C until analyzed. The residue was resuspended in 125 μl HPLC buffer A (50% methanol, 1% formic acid, 5 mM ammonium formate in water (JT Baker) and sonicated in a bath sonicator for 1 minute at 20°C. Analytes in a portion of the sample (10 μl) were then separated using liquid chromatography (Shimadzu, Nakagyo-ku, Kyoto, Japan) with a Luna 3 μm C18(2) 100Ǻ 50 × 2 mm column (Phenomenex, Torrance, CA, USA) and analyzed by tandem mass spectrometry on a 4000 QTRAP mass spectrometer (AB SCIEX, Framingham, MA, USA) in positive ion mode. The HPLC gradient was linear from buffer A to buffer B (10% isopropyl alcohol, 1% formic acid, 5 mM ammonium formate in methanol) over 1 minute at a flow rate of 0.4 ml/min. To wash the column, the gradient was repeated twice before equilibrating for the next sample. The transitions analyzed were 380.3/264.3 and 380.3/81.9 for endogenous S1P, and 366.2/93.0, 366.2/82.0 and 366.2/250.3 for internal standard with a dwell time of 15 milliseconds. Calibrators were in mouse plasma (C18 base D-erythro-sphingosine-1-phosphate, Avanti Polar Lipids). Between-day coefficient of variation was 7.7%. Pertinent instrument specific parameters were empirically derived and included curtain gas: 15, ion source voltage: 5000 V, emitter temperature: 550°C, desolvation gas 1: 20, desolvation gas 2: 70, collision gas: 6, entrance potential: 10, and collision cell exit potential: 10. Chromatographic data were analyzed using Analyst 1.4.2 (AB SCIEX) by summing transitions for each analyte.

### Creatine kinase (CK) assay

*mdx*^*4cv*^ mouse plasma samples were diluted 1:50 and total CK activity was measured by an enzymatic rate method at the clinical laboratory of the Department of Laboratory Medicine, University of Washington, using the Beckman Coulter instrument (Brea, CA, USA) as previously described [20]. Relative levels were then normalized to body weight.

### S1P injections

**Figure 5:**
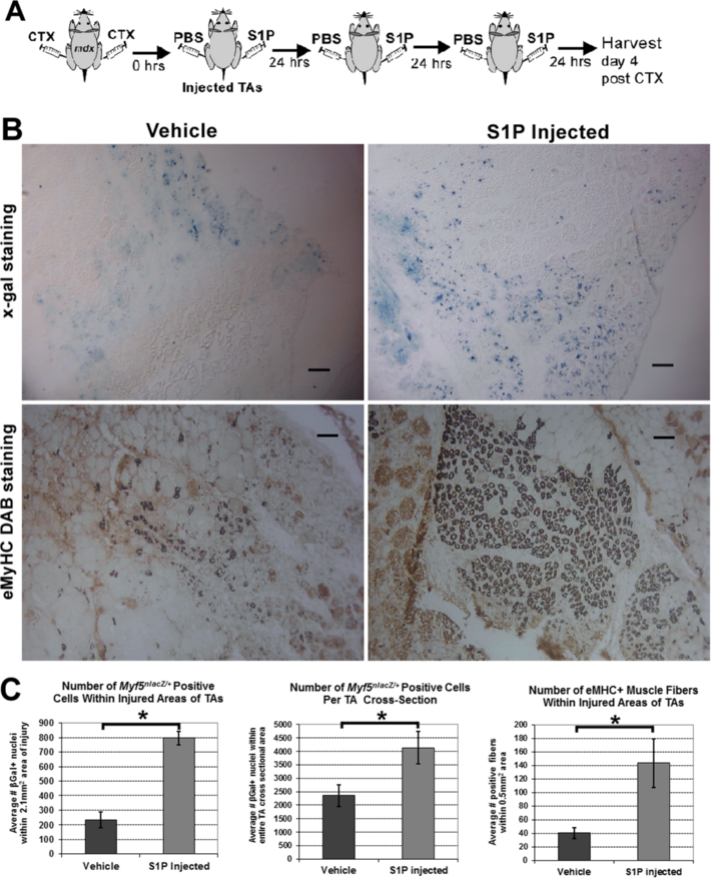
**Direct administration of S1P promotes muscle regeneration following acute injury. (A)** Experimental schematic of S1P and PBS (vehicle) injected daily for the first 72 hours into TAs of 3-MO *mdx*^*4cv*^*:Myf5*^*nlacZ/+*^ mice (n = 3, left TAs injected S1P, right TAs injected PBS) following CTX injury. **(B)** Top row: X-gal staining reveals an increased number of β-galactosidase+ nuclei at the sites of injury in S1P-treated TA muscles compared to vehicle controls. Bottom row: staining for eMyHC with DAB reveals a significant increase in the number of newly regenerated muscle fibers in S1P-treated TA muscles. Scale bars = 50 μm. **(C)** Left graph: quantification of β-galactosidase+ nuclei indicates the number of *Myf5*+ cells is significantly increased at the site of injury in S1P-treated compared to untreated muscles. Middle graph: a significant increase in β-galactosidase+ nuclei was also observed over the entire CSA of each S1P-treated TA muscle. Right graph: quantification of the number of eMyHC fibers within areas of regeneration was significantly greater with S1P treatment. *P <0.05 by student’s *t*-test. Error bars represent SEM. CSA, cross-sectional area; CTX, cardiotoxin; DAB, 3,3'-diaminobenzidine; eMyHC, embryonic myosin heavy chain; MO, month-old; S1P, sphingosine-1-phoshate; SEM, standard error of the mean; TA, tibialis anterior.

**Figure 6:**
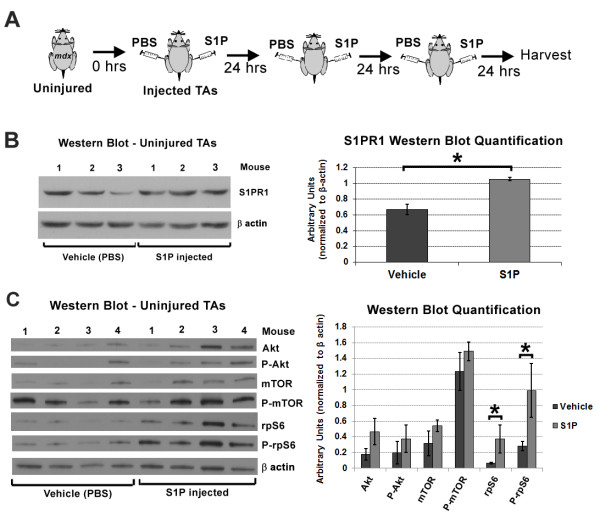
**Administration of S1P leads to increased levels of S1PR1 and P-rpS6 *in vivo*. (A)** Experimental schematic of S1P and PBS (vehicle) injected daily for the first 72 hours into TAs of uninjured *mdx*^*4cv*^ mice (n = 4, 2.5-MO, left TAs injected S1P, right TAs injected PBS). **(B)** Western blot analysis of injected TAs (n = 3, 2.5-MO *mdx*^*4cv*^) indicates that administration of S1P significantly increases S1PR1 levels. **(C)** Western blot analysis of injected TAs (n = 4, 2.5-MO *mdx*^*4cv*^) for total, and P-Akt, P-mTOR and P-rpS6, reveals that total and P-rpS6 were significantly higher with S1P treatment. Increased levels of total and P-rpS6 suggest that S1P administration promotes protein synthesis in *mdx* muscles. *P <0.05 by student’s *t*-test. Error bars represent SEM. MO, month-old; P-Akt, phosphorylated Akt; P-mTOR, phosphorylated mammalian target of rapamycin; P-rpS6, phosphorylated ribosomal protein S6; rpS6, ribosomal protein S6; S1P, sphingosine-1-phoshate; S1PR1, S1P receptor 1; SEM, standard error of the mean; TA, tibialis anterior.

**Figure 7:**
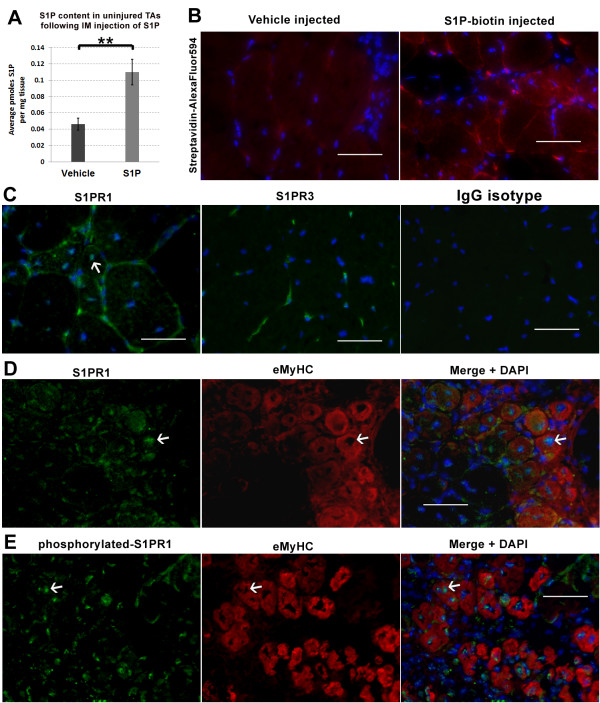
**Direct injection results in elevated S1P levels which correlate with the activation of receptor 1 in muscle fibers. (A)** To quantify the elevation of S1P following direct administration, we injected a single dose (same dose as Figure 5) of S1P in left TAs and vehicle in right TAs of uninjured *mdx*^*4cv*^ (n = 3, 11-MO) mice. TA muscles were harvested 15 minutes post injection for analysis by LC-MS/MS. Results indicate a significant elevation of S1P following direct injection. **(B)** To visualize the location of S1P following injection, biotinylated-S1P was injected in left TAs versus vehicle in right TAs of uninjured *mdx*^*4cv*^ mice (n = 2, 11-MO). Once more, TAs were harvested 15 minutes following injection. Staining with streptavidin conjugated to Alexa Fluor 594 reveals the presence of S1P-biotin around the perimeter of muscle fibers. **(C)** Staining of *mdx*^*4cv*^ TAs for S1PR1 and S1PR3 reveals S1PR1 is localized to the perimeter and perinuclear area (arrow) of muscle fibers (left photo). In contrast, staining for S1PR3 was mainly localized to the muscle vasculature (middle photo). Staining in parallel with an IgG isotype control for both antibodies shows the absence of non-specific staining (right graph). **(D)** Staining for S1PR1 in CTX-injured TAs (same tissue from Figure 5) reveals S1PR1 is present at the perimeter and perinuclear area of regenerating eMyHC+ fibers. **(E)** Staining for phosphorylated S1PR1 in the same *mdx*^*4cv*^ TAs was more prominent in the perinuclear area of eMyHC+ fibers, indicating the presence of active S1PR1 signaling in regenerating fibers. Scale bars = 50 μm. **P <0.005 by student’s *t*-test. Error bars represent SEM. CTX, cardiotoxin; eMyHC, embryonic myosin heavy chain; IgG, immunoglobulin G; LC-MS/MS, liquid chromatography-tandem mass spectrometry; MO, month-old; S1P, sphingosine-1-phoshate; S1PR1, S1P receptor 1; S1PR3, S1P receptor 3; SEM, standard error of the mean; TA, tibialis anterior.

Right and left TAs of three 3-MO male *mdx*^*4cv*^*:Myf5*^*nlacZ/+*^ were injured once more with 10 nM CTX (Figure 5). S1P (Enzo Life Sciences, Farmingdale, NY, USA; Calbiochem) preparation was undertaken according to manufacturer’s instructions. Briefly, S1P was dissolved in methanol (0.5 mg/ml) and aliquoted, then the solvent was evaporated with a stream of nitrogen to deposit a thin film on the inside of the tube. Prior to use, aliquots were resuspended in PBS with 4 mg/ml BSA (fatty acid free) to a concentration of 500 μM. Directly following CTX injection, 20 μl 500 μM S1P was injected in left TAs, daily until day 3 post injury, at which time animals were euthanized and muscles were harvested for freezing. Right TAs were injected with an equal volume of PBS with 4 mg/ml BSA as vehicle controls. In a separate experiment (Figure 6), TAs of four 2.5-MO female *mdx*^*4cv*^ were injected with S1P or vehicle under the same conditions stated above, in the absence of injury. AJ/SCID mice (n = 4, 9-MO, B6. Cg-*Dysf*^*prmd*^*Prkdc*^*scid*^/J) were also injected for 3 days with S1P or vehicle in TAs post CTX injury, following the same concentration and injection regimen used in *mdx*^*4cv*^. For measurement of S1P muscle content (Figure 7A) following intramuscular injections, 11-MO *mdx*^*4cv*^ (n = 3) were injected 20 μl 500 μM S1P in left TAs and 20 μl vehicle in right TAs. Muscles were harvested and frozen in liquid nitrogen 15 minutes post injection, and then processed using the aforementioned methods for analyzing S1P in muscle by LC-MS/MS. For injection of biotinylated-S1P, TAs from 11-MO *mdx*^*4cv*^ (n = 2) were injected intramuscularly with 20 μl 500 μM S1P-biotin or vehicle (Echelon Biosciences, Salt Lake City, UT, USA). TAs were harvested and frozen in OCT compound 15 minutes following injection.

### Mouse histology and immunohistochemistry

All mouse muscles were frozen directly in OCT compound with liquid nitrogen cooled in isopentane and sectioned 8 μm thick. Tissue for X-gal staining was fixed for 10 minutes with 2% formaldehyde/0.2% glutaraldehyde and incubated overnight at 37°C with staining buffer (PBS with 1 mg/ml X-gal, 5 mM potassium ferricyanide, 5 mM potassium ferrocyanide, 2 mM CaCl_2_ (all from Fisher Scientific)). Picrosirius red with fast green, hematoxylin and eosin, and Oil Red O staining were conducted following established protocols [21]. Fibrosis was quantified as percentage of area stained red within each 20 × field analyzed using ImageJ v1.40 or Adobe Photoshop CS2 (San Jose, CA, USA). For evaluating fibrosis, the mean value from three separate sections (200 μm apart in longitudinal distance) were analyzed from each muscle and used to calculate the overall mean for each muscle group outlined in the x-axis of Figure 1D. Lipid accumulation was quantified with the ImageJ cell counter plugin by counting fatty infiltrates in montages (stitched from 10 × photos) covering the entire CSA of each muscle. Muscles injected with S1P-biotin or vehicle were cut 8 μm thick, fixed for 5 minutes with 4% formaldehyde, and then stained with streptavidin conjugated to Alexa Fluor 594 (Life Technologies, Carlsbad, CA, USA) at 1:1000 in PBS and 1% BSA for 1 hour.

### Immunohistological staining

Staining was undertaken using freshly frozen *mdx*^*4cv*^ muscles. Pax7 staining was performed as outlined by Clever *et al*. [22] with slight modification. Sections were fixed overnight in 4% formaldehyde (from paraformaldehyde powder) at 4°C. Following fixation, antigen retrieval was performed with 10 mM citrate buffer (with 0.05% Tween 20 at pH 6.0) warmed in a water bath at 90°C for 20 minutes. Slides were then permeated with ice cold methanol for 5 minutes at room temperature. Streptavidin/biotin blocking (Vector Laboratories, Burlingame, CA, USA) was performed according to manufacturer’s instructions. Staining was undertaken using the Mouse on Mouse (MOM) Kit (Vector Laboratories) with immunoglobulin G (IgG) blocking for 5 hours at 4°C prior to addition of mouse monoclonal anti-Pax7 (clone PAX7, R&D Systems, Minneapolis, MN, USA) diluted at 1:20 and incubated overnight at 4°C. Biotinylated anti-mouse secondary was supplied with and used as prescribed by MOM Kit instructions. Streptavidin conjugated to Alexa Fluor 488 (Life Technologies) was added at 1:1000. As a negative control for Pax7 staining, a mouse IgG isotype was applied to separate ribbons and treated in parallel. For BS1 staining, muscles were initially fixed with 4% formaldehyde for 5 minutes at room temperature then stained with BS1 directly conjugated to fluorescein isothiocyanate (FITC), diluted at 1:400 in PBS with 1% BSA and applied for 1 hour at room temperature. Following BS1 staining, wheat germ agglutinin (WGA) directly conjugated to rhodamine was administered at 1:400 dilution as a counterstain for identifying myofibers. CD3e staining was undertaken in the same manner as BS1, using rat monoclonal anti-CD3e (clone 145-2C11, eBioscience, San Diego, CA, USA) at 1:100 dilution, followed by anti-rat IgG conjugated to Alexa Fluor 594 at 1:1000 dilution.

For laminin staining, tissue was also fixed with 2% formaldehyde for 5 minutes then treated with polyclonal rabbit anti-laminin (Sigma-Aldrich, St Louis, MO, USA) for 1 hour at 1:400 dilution in PBS and 1% BSA. Following washes, Alexa Fluor 488 conjugated goat anti-rabbit IgG (Life Technologies) was administered at 1:800 dilution for 1 hour. Controls omitting the primary antibody were included with all staining. For embryonic myosin heavy chain (eMyHC), tissue was first fixed with 2% formaldehyde for 5 minutes, treated with streptavidin/avidin blocking and blocked with IgG block from MOM Kit for 5 hours at 4°C. Following blockade, concentrated mouse anti-eMyHC (clone F1.652, received concentrated at 357 μg/ml IgG, Developmental Studies Hybridoma Bank (DSHB), University of Iowa, IA, USA) was administered at 1:400 dilution overnight at 4°C. The remainder of the staining was undertaken following MOM Kit staining instruction. 3,3'-diaminobenzidine (DAB) was used for visualizing and quantifying eMyHC fibers. For fluorescence, eMyHC was visualized using streptavidin conjugated to Alexa Fluor 594 used at 1:1000 dilution for 1 hour. For S1P receptor staining, slides were fixed with 4% formaldehyde for 5 minutes and stained with rabbit polyclonal IgG antibodies against S1PR1, S1PR3 (Cayman Chemical, Ann Arbor, MI, USA) and phosphorylated S1PR1 (raised against Thr236, Assay Biotechnology, Sunnyvale, CA, USA), all applied at a dilution of 1:200 for 2 hours. Following receptor staining, goat anti-rabbit IgG conjugated to Alexa Fluor 488 was added at 1:1000 for 1 hour. In parallel, we stained additional slides with rabbit polyclonal IgG isotype at the same final concentrations to exclude non-specific staining of these antibodies in *mdx*^*4cv*^ muscles.

Staining quantifications were all undertaken using ImageJ cell counter plugin. Calculations, statistics and graphs were generated with Microsoft Excel (Redmond, WA, USA). Bright field photographs were captured using either a Fisher Scientific Micromaster digital inverted or upright microscopes with Micron software. Fluorescent photographs were captured with a monochromatic camera using an Axiovert 200 microscope (Zeiss, Oberkochen, Germany). Individual fluorescent channels were colored and merged using Adobe Photoshop. Brightness contrast levels were adjusted to increase visibility and reduce background in most photographs.

### Western blot analysis

Tissue for western blot analysis was snap frozen in liquid nitrogen and subsequently homogenized. Freshly isolated TA muscles were harvested and snap frozen in liquid nitrogen prior to homogenization with disposable tissue grinders. Tissue was homogenized under liquid nitrogen then resuspended in lysis buffer containing 50 mM Tris–HCl (pH 7.4), 1 mM EDTA, 150 mM NaCl, 5 mM NaF, 0.25% (w/v) sodium deoxycholate, 2 mM NaVO_3_, 1% Triton X-100 (v/v), supplemented with complete protease inhibitor cocktail (Roche, Basel, Switzerland), and complete phosphatase inhibitor cocktails 1 and 2 (Sigma-Aldrich). Protein extracts were separated using Ready Gel Tris–HCl (BioRad, Hercules, CA, USA), 4 to 20% linear gradient and transferred to polyvinylidene fluoride (PVDF) membranes with a wet transfer system (BioRad). Membranes were blocked for 1 hour with Tris-buffered saline with 0.1% (v/v) Tween 20 containing 5% (w/v) BSA. For S1PR1 analysis, rabbit polyclonal anti-S1PR1 was used at a 1:500 dilution (Santa Cruz Biotechnology, Santa Cruz, CA, USA). Rabbit polyclonal antibodies were used to blot against phosphorylated (Thr308) Akt, total Akt, phosphorylated (Ser2448) mammalian target of rapamycin (mTOR), total mTOR, phosphorylated (Ser240/Ser244) rpS6, total rpS6 (1:1000, Cell Signaling Technology, Danvers, MA, USA) and β-actin (1:10000, Sigma-Aldrich). The signals were detected using an enhanced chemiluminescence kit (Millipore, Billerica, MA, USA) and CL-XPosure films (Thermo Scientific) were analyzed using ImageJ.

### Statistics

Student’s *t*-test was used to determine statistical significance for the majority of experiments. *P* values generated by analysis of variance (ANOVA) are specified in the text.

## Results

### Alterations of S1P regulation and content following IP injection of THI in *mdx* mice

To determine the effect of elevating S1P levels in dystrophic animals, we studied the effects of THI in the *mdx* mouse model for DMD [23,24]. Recently, Loh *et al*. (2012) showed that compared to wt, *mdx* muscles are in a state of S1P deprivation as they exhibit increased levels of the enzymes that degrade S1P (S1P lyase and S1P phosphatase 1) [8]. THI is a hydrophilic small molecule that increases S1P levels by inhibiting the lyase that irreversibly degrades S1P [11,25,26]. In turn, low doses of THI may be sufficient to cause mild lymphocytopenia but the presumable increase of S1P levels in muscle have not been reported [8,11]. To corroborate the effects of THI in *mdx*^*4cv*^ mice, we analyzed changes in lymphocytes before and after treatment, and measured S1P content in muscle (Figure 1). THI has low oral bioavailability; Bagdanoff *et al*. showed 10 to 12% bioavailability of THI when administered orally [10]. Thus we evaluated IP injections of THI as a parenteral delivery route for elevating systemic levels of THI. Peripheral blood was collected and analyzed before and 12 hours after two IP injections of THI (each injection was 250 μl 0.15 mg/ml THI, administered 6 hours apart). Following THI treatment, we observed a significant drop of all leukocytes except monocytes in *mdx*^*4cv*^ (n = 3, 1.5-MO) (Figure 1A). Of note, prior to treatment with THI, the total number of white blood cells and amount of individual leukocyte populations except monocytes, was significantly elevated in 1.5-MO *mdx*^*4cv*^ mice (n = 3) versus age-matched wt mice (n = 4). Interestingly, the number of platelets was also elevated twofold in *mdx*^*4cv*^ versus wt, but declined to near wt following THI administration (Additional file 1: Figure S1). This systemic effect in lymphocyte count indicates that THI functions efficiently when delivered systemically via IP injection. In addition, for short-term treatments, IP administration is desirable to ensure that all mice received the same dose. Thus for the majority of experiments described herein, we opted to administer THI via IP administration.

Loh *et al*. also demonstrated that following acute injury, the expression of S1P lyase increases in wt muscle [8]. Thus we analyzed the expression of enzymes that regulate S1P production and degradation following CTX injury in the *mdx* background with and without THI treatment. Right TA and quadriceps muscles were uninjured, while left counterparts were injured using CTX, a well characterized model of acute injury where initial muscle destruction is followed by a rapid myogenic response [27-30]. *mdx*^*4cv*^ mice (n = 6, 3.5-MO males) were injected IP immediately following CTX and thereafter five additional times during a 3-day period (for example 2 × IP injections per day) with either the previously used dose of THI or vehicle. For this analysis, muscles were harvested at day 4 post injury; the peak of myogenic gene expression following CTX-induced damage [28]. In the absence of THI, expression of the S1P lyase was significantly elevated following injury (Additional file 1: Figure S2A). Surprisingly, expression of S1P phosphatase 1 and lyase were greater in the injured muscles with THI treatment, suggesting a possible compensation in the S1P degradation pathways in response to the inhibition of the S1P lyase. Analogous to these results, expression levels of S1P kinase 1 were also increased with injury and at higher levels with THI (Additional file 1: Figure S2B). In contrast, the expression of S1P kinase 2 was only significantly elevated in the injured muscles from THI-treated animals. These results suggest that acute injury in *mdx*^*4cv*^ muscles induces upregulation of enzymes that regulate S1P metabolism. In turn, elevated expression of both S1P kinases with THI treatment may be beneficial for muscle regeneration in *mdx* mice. However, with THI treatment S1P phosphatase 1 and lyase expression were also greatly increased. Therefore we examined S1P content, to determine if THI treatment results in increased intramuscular S1P levels and in turn promotes muscle regeneration following CTX injury.

In order to determine if THI treatment results in increased intramuscular S1P levels, a second group of *mdx*^*4cv*^ animals was treated with THI or PBS (n = 6, 5-MO males), following the same dosing schedule (2 × IP injections per day for the first 3 days post CTX injury) and sacrificed at day 4 to analyze the efficacy of THI in increasing S1P levels (Figure 1B). In concordance with published work, treatment with THI increased S1P levels in spleen but not plasma (Figure 1B, Additional file 1: Figure S3A) [10,11]. S1P levels were also significantly increased in CTX-injured quadriceps from THI-treated animals (Figure 1B). This indicates that despite increased expression of S1P phosphatase 1 and lyase following injury, the counteracting increased expression of both S1P kinases results in elevated levels of intramuscular S1P. In addition, we also observed increased S1P levels in the uninjured TA muscles from mice treated with THI compared to vehicles. To examine if such extravascular increases of S1P correlated with a beneficial effect in dystrophic mice, we analyzed the level of plasma CK, which are elevated in humans and mice with muscular dystrophy activity in the same group of THI-treated *mdx*^*4cv*^ mice [31]. Results indicate a trending, but not statistically significant decline in CK activity levels in plasma collected on day 4 post injury from THI versus vehicle-treated mice (Additional file 1: Figure S3B).

### Reduction of dystrophic muscle pathology in acutely injured *mdx* muscles via administration of THI IP

Although young *mdx* mice exhibit robust muscle repair, regeneration becomes impaired with aging, resulting in muscle atrophy and dystrophy [3]. Therefore, in a third experiment, the effects of THI on histopathology were assessed in injured and uninjured muscles from two groups of aged *mdx*^*4cv*^ mice (n = 6, 11-MO females; n = 7, 16-MO males), to determine the effects of increasing levels of S1P in dystrophic animals at a stage of severe muscle wasting. Importantly, it has been reported that *mdx* females older than 6 months of age exhibit greater fibrosis than males [32]. Once more, right TA and quadriceps muscles were uninjured, while left counterparts were injured with CTX (Figure 2A). Regeneration following CTX injury is well orchestrated in normal muscle but impaired in older *mdx* mice [29]. Therefore in these studies we analyzed the muscles from 11- and 16-MO *md*x mice 18 days following CTX injury, a time point expected for non-diseased muscles to fully regenerate [28]. In the 16-MO mice, muscles were weighed immediately after collection and normalized to body weight (grams muscle weight over grams mouse weight). As expected, injured muscles were lighter than uninjured muscles in vehicle mice, an approximate weight loss greater than 20% (Additional file 1: Figure S4A). However, in the THI-treated mice the weight of injured quadriceps was similar to uninjured quadriceps (muscle weight ratio injured/uninjured approximates one), suggesting that THI treatment promotes muscle repair and protects from muscle loss following acute injury.

Fibrosis and fat deposition are both hallmarks of muscle wasting and dystrophic muscle pathology [32,33]. In addition, when regeneration is impaired, fibrosis and fat accumulate in place of muscle following acute injury [34,35]. Histological quantification revealed that THI treatment reduced accumulation of both fibrosis and fat deposition following acute injury in quadriceps and TA muscles (Figure 2B,C). Results for lower fibrosis were confirmed by third party hydroxyproline analysis of injured TAs from 16-MO animals (Additional file 1: Figure S4B). Interestingly, fibrosis was also significantly lower in uninjured TAs of 11-MO females, which correlates with the capacity of THI to elevate S1P levels in uninjured TAs (Figure 1B, Additional file 1: Figure S5). Although only left TAs and quadriceps were injected with CTX, fibrosis accumulation in uninjured muscles was likely elevated as mice disuse injured limbs and bear most of the use/weight on the uninjured contralateral limb. Therefore, the differences observed in uninjured TAs are likely due to reductions in the amount of fibrotic deposition that would otherwise accumulate without THI treatment, since it is unlikely THI can reverse already accrued fibrosis. Along with lower fibrosis observed in injured muscles, the overall morphology appeared more organized with THI treatment compared to vehicle-treated animals (Figure 2C). In addition, the number of EBD-positive fibers, an indicator of muscle fiber damage, was lower in injured 11-MO muscles and significantly reduced in uninjured 11-MO quadriceps (Additional file 1: Table S1) [12,36]. In these muscles the number of centrally nucleated fibers was comparable between THI and vehicle-treated animals (Additional file 1: Figure S6).

To test whether THI-treated mice show decreased fat deposition in injured muscles, we quantified the fat deposits within entire cross-sections of THI and vehicle-treated muscles (Figure 2D). The ratio of fat deposits between injured and uninjured contralateral muscles was then compared to THI and vehicle-treated mice (Figure 2E). This analysis indicates that THI significantly reduced fat deposition resulting from injury in 11-MO female TAs and 16-MO male quadriceps. These results demonstrate that THI treatment reduces injury-induced fat deposition and fibrosis in *mdx* muscles.

Further analysis of THI-treated *mdx*^*4cv*^ mice revealed an increase in muscle fiber size in quadriceps (Figure 3A). Although *mdx* mice undergo muscle hypertrophy as compared to wild type, we observed a significant increase in the minimum fiber diameter with THI treatment in diaphragms, and in both uninjured and injured quadriceps of 11-MO mice (Figure 3B,C and Additional file 1: Figure S7) [37]. Uninjured quadriceps of THI-treated 16-MO males also showed a significant increase in fiber size (Figure 3D). In summary, 3 days of THI treatment is sufficient to increase muscle fiber size in older *mdx* mice.

To assess if increases in muscle fiber size observed with THI treatment are accompanied by an increase in the number of satellite cells, we quantified the number of Pax7+ cells. Within skeletal muscle, Pax7 is specifically expressed by satellite cells, which have been reported to decline in older *mdx*^*4cv*^ muscles [38-40]. As expected, few satellite cells (Pax7+ nuclei) were visible in cross-sections of 11-MO *mdx* muscles. However, there was a significant increase in the mean number of Pax7+ nuclei, collectively in limb muscles (TAs and quadriceps) from THI-treated 11-MO animals (Additional file 1: Figure S8).

S1P is a potent angiogenic factor [41-43]. Thus we studied the effects of THI treatment on the skeletal muscle microvasculature. We quantified the number of vessels using BS1, a lectin that highlights endothelial cells [44]. In contrast to the increase in Pax7+ cells, we did not observe an increase in BS1+ vessels in injured 11-MO TA muscles. Quantitative RT-PCR analysis of endothelial related genes *eNO*S and *CD31* in 5-MO *mdx*^*4cv*^ TA muscles at day 4 post injury, show no significant difference in the levels of expression of these endothelial associated genes in THI treatment compared to vehicle (Additional file 1: Figure S9). This suggests that THI benefits on muscle repair do not depend on increasing microvasculature density.

### THI treatment elevates isometric force in acutely injured *mdx* EDL muscles

To assess if increasing S1P levels promotes dystrophic muscle function, in a fourth experiment we conducted myography analysis following longer treatment with THI. For this experiment, another group of *mdx* mice (male 4.75- to 5-MO *C57BL/10ScSn-Dmd*^*mdx/J*^*)* was injured and treated with daily IP injections using the same THI dose and injection interval, for 14 consecutive days; the maximum duration for IP administration allowed by our approved animal protocol. Animals were treated with THI (n = 10) or vehicle (n = 9) for 14 days following injury, and analyzed between day 15 and 19 (Figure 4A). EDL muscles from injured and uninjured contralateral limbs were analyzed for isometric specific force; a physiological measurement of muscle force that is reduced with muscular dystrophy in mice and humans [18,45,46].

To assess if the EDL is damaged as a consequence of CTX injection in the TA, we injured and analyzed a separate group of *mdx* mice (n = 4) 12 hours post injury. For this fifth experiment, CTX injections included India ink to label needle penetration [47]. To assess muscle fiber damage, a consequence of CTX injury, animals were injected IP with EBD immediately following CTX injection. The presence of EBD indicates EDL muscles are damaged. However, EDL damage is not due to direct penetration by the needle since India ink was only present in the CTX-injected TA muscles (Additional file 1: Figure S10).

Force frequency analysis revealed a significantly higher specific force by EDL muscles isolated from injured limbs of THI-treated mice (Figure 4B). These values were similar to EDL muscles isolated from contralateral uninjured limbs, indicating that THI prevented wasting and preserved muscle function following acute injury (Figure 4B). However, the specific force observed after THI treatment was still lower than wt control animals (Figure 4C). Two weeks of THI treatment was not sufficient to improve specific force in uninjured EDL muscles. However, as shown in Figure 1B, the THI dose of 0.75 μg/day used for all our experiments does not significantly raise S1P levels in all uninjured *mdx* muscles. In addition, although peripheral lymphocytes declined with THI (Figure 1A), we did not observe a decline of CD3e+ T-cells present in the diaphragm following 2 weeks of THI (Additional file 1: Figure S11) [48]. Therefore, it is plausible that a higher dose of THI is required to sufficiently elevate S1P levels needed to improve specific force in uninjured *mdx* muscles. However, since THI is insoluble in PBS at higher concentrations and has low oral bioavailability, we chose to directly study the effects of high levels of S1P on uninjured *mdx* muscles *ex vivo*. For this experiment, EDLs from uninjured and untreated *mdx* mice were analyzed following incubation with 10 μM S1P [16]. Analysis of the maximal specific force indicates that direct administration of S1P significantly increases force output in uninjured *mdx* muscle (Figure 4D). Such results indicate that treatment with high concentrations of S1P can promote functional improvement of dystrophic muscles.

Overall, reduction in fibrosis and fat deposition, and increase in myofiber size and satellite cell numbers, indicate that elevating S1P levels, pharmacologically or by direct administration, has a profound benefit in dystrophic muscle repair and function.

### Direct administration of S1P promotes muscle regeneration in *mdx* mice following CTX injury

S1P is essential for satellite cell turnover, myoblast differentiation and muscle regeneration in non-diseased mice, and more recently shown to promote satellite cell activation in *mdx* muscle [4,5,8,47]. To determine if the increase in satellite cell number observed in the THI-treated muscles was a result of increased S1P muscle content, we examined the effects of direct S1P administration following CTX-induced acute injury in dystrophic muscles. In order to identify satellite cells and their progeny, we utilized *mdx*^*4cv*^:*Myf5*^*nlacz/+*^ mice carrying the nuclear *lacZ* reporter driven by the endogenous *Myf5* gene, a marker of myogenic cells [49-51]. CTX was applied to both TA muscles (n = 3, 3-MO *mdx*^*4cv*^*:Myf5*^*nlacz/+*^ males), then S1P was immediately injected intramuscularly into left TAs and a vehicle control into right TAs. Injections were repeated daily for the first 72 hours following injury and TAs were harvested on day 4 post injury, directly following the peak of injury-induced myogenic cell proliferation for analysis of *Myf5+* nuclei (Figure 5A) [28]. S1P-treated muscles showed a dramatic, fourfold increase in the number of *Myf5*+ nuclei in areas with severe CTX damage compared to vehicle controls (Figure 5B top row and 5C left graph). Furthermore, a significant increase in the number of *Myf5*+ nuclei was observed over the entire CSA of S1P-treated TAs (Figure 5C middle graph, Additional file 1: Figure S12). These data demonstrate that S1P treatment increases the number of myogenic cells in *mdx* muscles following injury and suggests that S1P promotes satellite cell proliferation *in vivo.*

We then determined whether the increase in myogenic cells promotes dystrophic muscle repair by staining for eMyHC, a marker of regenerating muscle fibers [27]. In concurrence with the rise of *Myf5*+ myogenic cells, a 3.6 fold increase in the number of eMyHC+ fibers was observed in S1P-treated TAs (Figure 5B bottom row, 5C right graph). This increase in eMyHC+ fibers, corresponded with elevated numbers of centrally nucleated muscle fibers in the injured regions of S1P-treated muscles (Additional file 1: Figure S13A). Furthermore, the size of regenerating myofibers in S1P-treated TAs was significantly greater, as indicated by the minimum diameter quantified for the largest eMyHC+ fibers (Additional file 1: Figure S13B). Collectively, these data show that local administration of S1P promotes dystrophic muscle repair by improving satellite cell response and contribution to muscle fiber regeneration.

### S1P directly acts on *mdx* muscle fibers, and elevates levels of total and phosphorylated S1PR1

In mammals there are five S1P receptors that share homology to G-protein coupled receptors [52]. It has been recently reported that S1P receptor 2 (S1PR2) is specifically activated in myogenic cells and that downstream effectors of S1P action in satellite cells include components of the JAK-STAT signaling pathway [8]. In contrast, our results and others, of exogenous S1P treatment resulting in increased EDL force, suggests that S1P also acts directly on muscle fibers [16]. The amount of exogenous S1P added in the bath was super-physiological and thus we measured S1P muscle levels following intramuscular injection of S1P. In this experiment, left TAs from *mdx*^*4cv*^ mice (n = 3, 11-MO) were injected with the same dose of S1P as the *mdx*^*4cv*^*:Myf5*^*nlacz/+*^ mice depicted in Figure 5A, while contralateral TAs received the same vehicle. In contrast to the previous experiment depicted in Figure 5A, TA muscles were injected in the absence of injury and were harvested for S1P analysis 15 minutes post injection (Figure 6A); the same time used for S1P incubation prior to EDL force measurement shown in Figure 4D. Results indicate that within this timeframe, intramuscular injection of S1P does significantly increase S1P levels in *mdx* muscle (Figure 6A).

To directly observe where S1P binds in the muscle, a separate group of *mdx*^*4cv*^ (n = 2, 11-MO) were injected with the same amount of biotinylated-S1P in left and vehicle in right TAs. Once more, TAs were harvested 15 minutes post injection for histological visualization of S1P. Staining with streptavidin conjugated to Alexa Fluor 594 reveals that biotinylated-S1P is present in many cells, but particularly localized to the perimeter of muscle fibers (Figure 5B). Among the three S1P receptors (S1PR1, S1PR2, S1PR3) expressed in muscle, S1PR3 and S1PR1 are the most abundant in wt muscle [5]. Importantly, expression of these three S1P receptors is reduced in *mdx* muscle cells, especially S1PR1, which shows more than five fold reduction in relative mRNA levels (Additional file 1: Figure S14). Staining of *mdx*^*4cv*^ muscles (3.5-MO) for S1PR1 and S1PR3, reveals that S1PR1 is present at the perimeter of muscle fibers and myonuclei, whereas S1PR3 appears localized to the vasculature (Figure 6C). S1PR1 is a G protein-coupled receptor (GPCR) that can be activated via phosphorylation, resulting in translocation to the endosomal compartment and/or the perinuclear compartment [53-55]. Therefore, perinuclear localization of S1PR1 suggested that in response to S1P treatment, receptor 1 signaling is activated in *mdx*^*4cv*^ muscle fibers. To evaluate the presence of active S1PR1 signaling during muscle fiber regeneration, we surveyed the same CTX-injured muscles depicted in Figure 5A for the presence of phosphorylated S1PR1. Results indicate S1PR1 is localized around the perimeter of muscle fibers and intracellularly near or within the myonuclei (perinuclear) of newly regenerated eMyHC+ fibers (Figure 6D). In parallel, we observed more concentrated staining for phosphorylated S1PR1 localized perinuclearly and less so around the perimeter of eMyHC+ fibers (Figure 6E). These results indicate that S1PR1 signaling is active in regenerating muscle fibers and suggests that the beneficial actions that S1P exerts on *mdx* muscle fibers may be mediated through S1PR1.

### S1P administration correlates with increased levels of S1PR1 and P-rpS6, an indicator of protein synthesis

S1PR1 has been implicated in myoblast proliferation and shown to steadily increase during the course of regeneration in non-diseased muscle [4,5]. Therefore to gain more insight on the potential action that S1P exerts via S1PR1 in dystrophic muscle, we injected S1P in uninjured TAs of *mdx*^*4cv*^ (n = 3, 2.5-MO), and quantified the level of S1PR1 and some downstream effectors (Figure 7A) [56]. In turn, S1P treatment resulted in significantly elevated levels of S1PR1 in *mdx*^*4cv*^ TAs (Figure 7B). In a separate experiment, we injected S1P in left TAs and vehicle in right TAs of *mdx*^*4cv*^ (n = 3, 10-MO), following the same dose and experimental design (three injections, one per day, harvest on day 4), and analyzed TA muscles for phosphorylated S1PR1. Results from this experiment show that phosphorylated S1PR1 is also significantly elevated with S1P treatment (Additional file 1: Figure S15).

A result of S1P injection was larger eMyHC+ fibers that were positive for phosphorylated S1PR1 (Figure 6E, Additional file 1: Figure S13B). Therefore, we examined if elevated S1PR1 levels corresponded with known regulators of cell size and protein synthesis; Akt, mTOR, S6 kinase and rpS6. S1P-induced hypertrophy has been described in cultured cardiomyocytes, which was accompanied by activation of Akt and S6 kinase [57]. In addition, S1PR1 activation of S6 kinase via a Gi-dependent pathway has been reported in vascular smooth muscle cells [56]. Akt and mTOR signaling via S6 kinase, an activator of rpS6 implicated in protein synthesis, has been described as sufficient to induce skeletal muscle hypertrophy [58-60]. Therefore, we evaluated if direct injection of S1P induces activation of these pathways in uninjured TA muscles of *mdx*^*4cv*^ mice (n = 4, 2.5-MO). Western blot analysis of TA muscles injected for 3 days with S1P (Figure 7A) revealed that the levels of phosphorylated Akt (P-Akt) and mTOR (P-mTOR), though increased, were not significantly higher in S1P-treated muscles (Figure 7C). However, the levels of rpS6 and phosphorylated rpS6 (P-rpS6) were significantly increased with S1P treatment compared to control muscles, suggesting an increase in protein synthesis. Although a more detailed study is required to elucidate the role of S1P in skeletal muscle protein synthesis, our data suggest that S1P can activate muscle anabolic pathways in the *mdx* mouse.

### Direct administration of S1P promotes muscle regeneration in dysferlinopathy mice following acute injury

The role of dysferlin is currently unknown, but its absence in humans and mice results in chronic muscle wasting that primarily affects limb and girdle muscles [61-63]. Although dysferlinopathy is less severe than DMD [64], dysferlinopathy patients are often wheelchair bound between 30 and 40 years of age [65]. Much like DMD, muscles in humans and mice lacking functional dysferlin exhibit chronic atrophy, resulting in the accumulation of fibrosis and fat [66]. Therefore we tested the effects of S1P administration after CTX injury in a model of dysferlinopathy (AJ/SCID) to evaluate if the benefits of S1P are exclusive to the *mdx* background or can be applied to other muscle wasting diseases [67]. We followed the same experimental design outlined in Figure 5A, injecting left TAs of AJ/SCID mice (n = 4, 9-MO) with the same dose of S1P and vehicle in right TAs for 3 days following CTX injury. In contrast to the experiments in *mdx*^*4cv*^, we harvested TAs on day 6 post injury in order to also evaluate the onset of fibrosis. In accordance to the results observed in *mdx*, we observed improved muscle regeneration with the administration of S1P in AJ muscles. Specifically, we observed lower fibrosis and increased centrally nucleated fibers, as well as improved muscle architecture in the damaged regions of muscle with S1P administration (Additional file 1: Figure S16). These results indicate that approaches aimed at elevating muscle S1P may be beneficial to promote muscle regeneration in additional muscle wasting diseases.

### Longer-term treatment with THI shows a functional benefit in uninjured *mdx* muscle

**Figure 8:**
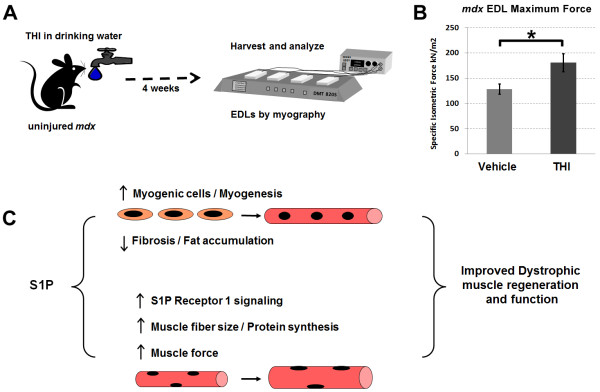
**Longer-term treatment with THI elevated muscle force in uninjured *mdx *EDL muscles. (A)** Experimental schematic outlining the treatment regimen. Beginning at 4 weeks of age, *mdx*^*4cv*^ mice (1-MO males) were treated for 4 weeks *ad libitum* with 50 mg/l THI (n = 4) or vehicle (n = 3) in drinking water. **(B)** Myography analysis of EDL muscles reveals a significant increase in maximal specific force with THI treatment. *P <0.05 by student’s *t*-test. Error bars represent SEM. **(C)** Summary of findings: S1P can act to not only promote myogenic cell activation and muscle repair, but also enhance muscle fiber size and force, possibly through S1PR1 mediated signaling. EDL, extensor digitorum longus; MO, month-old; S1P, sphingosine-1-phoshate; S1PR1, S1P receptor 1; SEM, standard error of the mean; THI, 2-acetyl-4(5)-tetrahydroxybutyl imidazole.

To this point we have largely examined the role of S1P in promoting muscle regeneration in acutely injured dystrophic muscles. Since long-term intramuscular injections of S1P are neither feasible nor practical (the injections also cause damage), we decided to revisit the use of THI for elevating S1P muscle content. Although our initial experiments with THI showed little benefit in uninjured *mdx* muscles, they were short-term and in older animals with severe pathology (Figures 2, 3), or adult animals (Figure 4) at a point when hypertrophy and robust regeneration compensate for degeneration in limb muscles [24,68,69]. Therefore, we examined longer-term treatment of THI in younger *mdx* mice at 4 weeks of age, a time point characterized by significant muscle degeneration prior to the compensatory period [70]. For this experiment, uninjured *mdx*^*4cv*^ animals were treated for 1 month, beginning at 4 weeks of age, with THI or vehicle in the drinking water (Figure 8A) [11]. At 8 weeks of age*,* we assessed the functional benefit of THI treatment by analyzing EDL specific force via myography. In turn, EDLs from THI-treated animals showed significantly greater specific force compared to vehicle-treated controls (Figure 8B). This data demonstrates that elevating S1P levels is beneficial for the chronic muscle injury that occurs early in muscular dystrophy.

## Discussion

We have shown that systemic administration of the pharmacological agent THI by IP injection to dystrophic *mdx* mice led to elevated levels of S1P in recovering injured muscle tissue, as well as a reduction of fibrosis and fat infiltration, both pathological indicators of muscle wasting (Figure 2). Additionally, systemic THI led to a significant increase in muscle fiber size and specific force of CTX-injured muscles (Figures 3 and 4). In turn, *ex vivo* administration of high levels of S1P resulted in specific force levels in uninjured *mdx* EDL muscles (Figure 4D). To pursue a better understanding of how elevated S1P reduces DMD pathology, we found that direct administration of S1P via intramuscular injection doubles muscle S1P content compared to the S1P levels reached with IP injections of THI. In addition, intramuscular S1P injections led to an increase in myogenic cells (*Myf5*+) and induced phosphorylation of S1PR1, which was particularly abundant in newly regenerating fibers (Figure 7, Additional file 1: Figure S15), as well as a significant increase in rpS6 and P-rpS6 levels (Figure 6). These results suggest that S1P not only works to activate myogenic precursors but also elevates protein synthesis in muscle fibers, potentially through S1PR1 mediated signaling (summarized in Figure 8C). In summary, THI/S1P administration led to improved regeneration and pathology, higher muscle specific force, an increase in the number of myogenic cells, and larger muscle fibers.

Our results indicate that S1P mediates satellite cell-dependent and muscle fiber-dependent effects on skeletal muscle. If amelioration of muscle wasting occurs through receptor-mediated signaling then S1P, elevated intracellularly via THI, must be exported to activate the S1P receptors. THI has been reported to inhibit the S1P lyase, an enzyme whose active site is on the cytoplasmic side of the endoplasmic reticulum. Therefore elevations of S1P levels mediated via THI inhibition of the S1P lyase presumably occur within the cytoplasm [71]. S1P may also act intracellularly before possible export to promote muscle wasting suppression. This alternative is supported by our work with *Drosophila*, which have no known S1P receptors [9], as well as by a recent report that showed S1P interacts directly, intracellularly, with histone deacetylases (HDACs) [72]. As HDAC inhibitors have been previously shown to suppress dystrophic phenotypes in *mdx* mice, the actions of S1P on the suppression of muscle wasting may occur in part through such mechanisms [73]. It has also been reported that reductions in HDAC activity result in an increase of follistatin, an inhibitor of myostatin, which may explain the amelioration of DMD pathology [74]. Our data support this possibility and suggest that the molecular mechanism for the suppression of muscle degeneration involves the anabolic pathways for muscle formation rpS6. These components have been shown to lie downstream of myostatin and insulin-like growth factor [75].

## Conclusion

Based on the work reported here, elevation of S1P may be a fruitful strategy for ameliorating the pathology manifested in patients afflicted with DMD and possibly other muscle wasting diseases (for example dysferlinopathy). Therapies based on promoting S1P levels in dystrophic muscle have the potential to improve pathology by promoting satellite cell and anabolic-mediated regeneration. An obvious candidate for a small molecule therapeutic is THI. Our work has shown that short-term treatment of THI has significant efficacy in increasing regenerative capacity in the *mdx* mouse following acute muscle injury, while longer treatment can improve muscle function in younger uninjured *mdx* muscle. Moreover, significant increases in muscle fiber size have been suggested as a viable approach in overcoming dystrophic muscle damage by promoting strength and function [76]. Additionally, there are other THI derivatives with increased oral bioavailability that may be more effective at increasing and maintaining high intramuscular S1P levels in long-term treatments, which was necessary for functional improvement of uninjured EDL muscles [10]. Alternatively there are inhibitors of lipid phosphate phosphatases and/or S1P phosphatases that may also increase intramuscular S1P levels [10,77]. In addition, there are specific S1P receptor agonists (for example FTY720) that are currently FDA approved or in clinical trials [78,79]. Based on our present results and those of others, future studies focused on S1P-based therapeutics for the treatment of DMD and related myopathies are warranted.

## Abbreviations

ANOVA: Analysis of variance; BSA: Bovine serum albumin; CK: Creatine kinase; CSA: Cross-sectional area; CTX: Cardiotoxin; DAB: 3,3'-diaminobenzidine; DAPI: 4',6-diamidino-2-phenylindole; DMD: Duchenne muscular dystrophy; DSHB: Developmental Studies Hybridoma Bank; EBD: Evans Blue dye; EDL: Extensor digitorum longus; EDTA: Ethylenediaminetetraacetic acid; eMyHC: Embryonic myosin heavy chain; FDA: Food and Drug Administration; FITC: Fluorescein isothiocyanate; GPCR: G protein-coupled receptor; HDAC: Histone deacetylase; HPLC: High performance liquid chromatography; IgG: Immunoglobulin G; IP: Intraperitoneal; LC-MS/MS: Liquid chromatography-tandem mass spectrometry; Lf: Fiber length; LPP3: Lipid phosphate phosphatase 3; LPP4: Lipid phosphate phosphatase 4; MO: Month-old; MOM: Mouse on Mouse; mTOR: Mammalian target of rapamycin; OCT: Optimal cutting temperature; P-Akt: Phosphorylated Akt; PBS: Phosphate-buffered saline; PCR: Polymerase chain reaction; P-mTOR: Phosphorylated mTOR; P-rpS6: Phosphorylated rpS6; PVDF: Polyvinylidene fluoride; rpS6: Ribosomal protein S6; RT-PCR: Reverse transcription polymerase chain reaction; S1P: Sphingosine-1-phoshate; S1PR1: S1P receptor 1; S1PR2: S1P receptor 2; S1PR3: S1P receptor 3; S1PR4: S1P receptor 4; SEM: Standard error of the mean; TA: Tibialis anterior; THI: 2-acetyl-4(5)-tetrahydroxybutyl imidazole; WGA: Wheat germ agglutinin; wt: wild type.

## Competing interests

Authors disclose the filing of a patent on S1P promoting therapies for muscular dystrophy. However, authors have no received reimbursements, shares/stocks, fees, funding, or salary from any organization that may in any way gain or lose financially from the publication of this manuscript. Authors declare no other financial competing interests.

## Authors’ contributions

NI and MP designed and conducted experiments, and wrote and edited the manuscript. ALH, TLD, JQ, KAF, ANH and MS conducted experiments. JSC, HRB and MR contributed to the experimental designs, editing and writing of the manuscript. All authors read and approved the final manuscript.

## Supplementary Material

Additional file 1: Figure S1 Treatment with THI lowers *mdx* plasma platelet levels. **Figure S2.** THI alters the expression of S1P regulatory gene in *mdx* muscle. **Figure S3.** THI does not alter S1P plasma levels but lowers plasma CK activity. **Figure S4.** Muscle weight is preserved and hydroxy proline is reduced in injured muscles from THI treated *mdx* mice. **Figure S5.** Fibrosis is lower in uninjured *mdx* TA muscles with THI treatment. **Figure S6.** The number of centrally nucleated muscle fibers does not change with THI. **Figure S7.** Diaphragm muscle fibers size increases with THI treatment. **Figure S8.** THI treated *mdx* mice have an elevated number of Pax7+ satellite cells. **Figure S9.** The microvasculature of *mdx* muscles did not increase with THI. **Figure S10.** CTX injected in the TA reaches and also damages the EDL. **Figure S11.** THI treatment did not reduce T-cells in *mdx* diaphragms. **Figure S12.** Montages covering entire cross-sectional areas of each TA from S1P and vehicle treated *mdx4CV:Myf5nlacZ/+* animals, were created by combining individual 10x photos. **Figure S13. (*A*) **Quantification of centrally nucleated muscle fibers from the same injured TAs presented in Figure 5, coincides with the number of newly regenerated fibers (eMyHC+ fibers) observed in S1P injected TA muscles. **(*B*)** Quantification of the minimum diameter of the largest eMyHC+ myofibers represented in Figure 5, indicates a significant increase in regenerated fiber size with S1P treatment. **Figure S14.** The expression of S1P receptors is reduced in *mdx* muscle cells. **Figure S15.** Direct S1P administration results in elevated levels of phosphorylation S1PR1 in *mdx* muscles. **Figure S16.** S1P promotes muscle regeneration in the A/J mouse model of dysferlinopathy. **Table S1.** Average number of Evans Blue+ muscle fibers within each muscle group.
Click here for file

## References

[B1] Deconinck N Dan B Pathophysiology of duchenne muscular dystrophy: current hypotheses Pediatr Neurol 2007 36 1 7 10.1016/j.pediatrneurol.2006.09.016 17162189

[B2] Mendell JR Rodino-Klapac LR Malik V Molecular therapeutic strategies targeting Duchenne muscular dystrophy J Child Neurol 2010 25 1145 1148 10.1177/0883073810371005 20498331 PMC3674570

[B3] Palmieri B Tremblay JP Daniele L Past, present and future of myoblast transplantation in the treatment of Duchenne muscular dystrophy Pediatr Transplant 2010 14 813 819 10.1111/j.1399-3046.2010.01377.x 20963914

[B4] Rapizzi E Donati C Cencetti F Nincheri P Bruni P Sphingosine 1-phosphate differentially regulates proliferation of C2C12 reserve cells and myoblasts Mol Cell Biochem 2008 314 193 199 10.1007/s11010-008-9780-y 18454302

[B5] Danieli-Betto D Peron S Germinario E Zanin M Sorci G Franzoso S Sandona D Betto R Sphingosine 1-phosphate signaling is involved in skeletal muscle regeneration Am J Physiol 2010 298 C550 C558 10.1152/ajpcell.00072.2009 20042733

[B6] Bernacchioni C Cencetti F Blescia S Donati C Bruni P Sphingosine kinase/sphingosine 1-phosphate axis: a new player for insulin-like growth factor-1-induced myoblast differentiation Skelet Muscle 2012 2 15 10.1186/2044-5040-2-15 22788716 PMC3439699

[B7] Bruni P Donati C Pleiotropic effects of sphingolipids in skeletal muscle Cell Mol Life Sci 2008 65 3725 3736 10.1007/s00018-008-8236-6 18668202 PMC11131905

[B8] Loh KC Leong WI Carlson ME Oskouian B Kumar A Fyrst H Zhang M Proia RL Hoffman EP Saba JD Sphingosine-1-phosphate enhances satellite cell activation in dystrophic muscles through a S1PR2/STAT3 signaling pathway PLoS One 2012 7 e37218 10.1371/journal.pone.0037218 22606352 PMC3351440

[B9] Pantoja M Fischer KA Ieronimakis N Reyes M Ruohola-Baker H Genetic elevation of Sphingosine 1-phosphate suppresses dystrophic muscle phenotypes in Drosophila Development 2013 140 136 146 10.1242/dev.087791 23154413 PMC3513996

[B10] Bagdanoff JT Donoviel MS Nouraldeen A Tarver J Fu Q Carlsen M Jessop TC Zhang H Hazelwood J Nguyen H Baugh SD Gardyan M Terranova KM Barbosa J Yan J Bednarz M Layek S Courtney LF Taylor J Digeorge-Foushee AM Gopinathan S Bruce D Smith T Moran L O'Neill E Kramer J Lai Z Kimball SD Liu Q Sun W Inhibition of sphingosine-1-phosphate lyase for the treatment of autoimmune disorders J Med Chem 2009 52 3941 3953 10.1021/jm900278w 19489538

[B11] Schwab SR Pereira JP Matloubian M Xu Y Huang Y Cyster JG Lymphocyte sequestration through S1P lyase inhibition and disruption of S1P gradients Science (New York, NY 2005 309 1735 1739 10.1126/science.1113640 16151014

[B12] Matsuda R Nishikawa A Tanaka H Visualization of dystrophic muscle fibers in mdx mouse by vital staining with Evans blue: evidence of apoptosis in dystrophin-deficient muscle J Biochem 1995 118 959 964 10.1093/jb/118.5.959 8749313

[B13] Ieronimakis N Balasundaram G Rainey S Srirangam K Yablonka-Reuveni Z Reyes M Absence of CD34 on murine skeletal muscle satellite cells marks a reversible state of activation during acute injury PLoS One 2008 5 e10920 20532193 10.1371/journal.pone.0010920 PMC2880004

[B14] Grabski AD Shimizu T Deou J Mahoney WM Jr Reidy MA Daum G Sphingosine-1-phosphate receptor-2 regulates expression of smooth muscle alpha-actin after arterial injury Arterioscler Thromb Vasc Biol 2009 29 1644 1650 10.1161/ATVBAHA.109.191965 19608972 PMC2746263

[B15] Au CG Butler TL Sherwood MC Egan JR North KN Winlaw DS Increased connective tissue growth factor associated with cardiac fibrosis in the mdx mouse model of dystrophic cardiomyopathy Int J Exp Pathol 2011 92 57 65 10.1111/j.1365-2613.2010.00750.x 21121985 PMC3052757

[B16] Danieli-Betto D Germinario E Esposito A Megighian A Midrio M Ravara B Damiani E Libera LD Sabbadini RA Betto R Sphingosine 1-phosphate protects mouse extensor digitorum longus skeletal muscle during fatigue Am J Physiol 2005 288 C1367 C1373 10.1152/ajpcell.00246.2004 15659717

[B17] Gregorevic P Plant DR Leeding KS Bach LA Lynch GS Improved contractile function of the mdx dystrophic mouse diaphragm muscle after insulin-like growth factor-I administration Am J Pathol 2002 161 2263 2272 10.1016/S0002-9440(10)64502-6 12466140 PMC1850914

[B18] Gregorevic P Plant DR Lynch GS Administration of insulin-like growth factor-I improves fatigue resistance of skeletal muscles from dystrophic mdx mice Muscle Nerve 2004 30 295 304 10.1002/mus.20082 15318340

[B19] Baker DL Desiderio DM Miller DD Tolley B Tigyi GJ Direct quantitative analysis of lysophosphatidic acid molecular species by stable isotope dilution electrospray ionization liquid chromatography-mass spectrometry Anal Biochem 2001 292 287 295 10.1006/abio.2001.5063 11355863

[B20] Oliver IT A spectrophotometric method for the determination of creatine phosphokinase and myokinase Biochem J 1955 61 116 122 13260184 10.1042/bj0610116 PMC1215753

[B21] Kiernan J Histological and Histochemical Methods: Theory and Practice 2008 4 New York: Cold Spring Harbor Laboratory Press

[B22] Clever JL Sakai Y Wang RA Schneider DB Inefficient skeletal muscle repair in inhibitor of differentiation knockout mice suggests a crucial role for BMP signaling during adult muscle regeneration Am J Physiol 2010 298 C1087 C1099 10.1152/ajpcell.00388.2009 PMC2867391 20181926

[B23] Chapman VM Miller DR Armstrong D Caskey CT Recovery of induced mutations for X chromosome-linked muscular dystrophy in mice Proc Natl Acad Sci USA 1989 86 1292 1296 10.1073/pnas.86.4.1292 2919177 PMC286674

[B24] Pastoret C Sebille A mdx mice show progressive weakness and muscle deterioration with age J Neurol Sci 1995 129 97 105 10.1016/0022-510X(94)00276-T 7608742

[B25] Zhou J Saba JD Identification of the first mammalian sphingosine phosphate lyase gene and its functional expression in yeast Biochem Biophys Res Commun 1998 242 502 507 10.1006/bbrc.1997.7993 9464245

[B26] Mendel J Heinecke K Fyrst H Saba JD Sphingosine phosphate lyase expression is essential for normal development in Caenorhabditis elegans J Biol Chem 2003 278 22341 22349 10.1074/jbc.M302857200 12682045

[B27] d’Albis A Couteaux R Janmot C Roulet A Mira JC Regeneration after cardiotoxin injury of innervated and denervated slow and fast muscles of mammals. Myosin isoform analysis E J Biochem 1988 174 103 110 10.1111/j.1432-1033.1988.tb14068.x 3371354

[B28] Yan Z Choi S Liu X Zhang M Schageman JJ Lee SY Hart R Lin L Thurmond FA Williams RS Highly coordinated gene regulation in mouse skeletal muscle regeneration J Biol Chem 2003 278 8826 8836 10.1074/jbc.M209879200 12477723

[B29] Mouisel E Vignaud A Hourde C Butler-Browne G Ferry A Muscle weakness and atrophy are associated with decreased regenerative capacity and changes in mTOR signaling in skeletal muscles of venerable (18-24-month-old) dystrophic mdx mice Muscle Nerve 2010 41 809 818 10.1002/mus.21624 20151467

[B30] Ieronimakis N Balasundaram G Rainey S Srirangam K Yablonka-Reuveni Z Reyes M Absence of CD34 on murine skeletal muscle satellite cells marks a reversible state of activation during acute injury PLoS One 2010 5 e10920 10.1371/journal.pone.0010920 20532193 PMC2880004

[B31] Glesby MJ Rosenmann E Nylen EG Wrogemann K Serum CK, calcium, magnesium, and oxidative phosphorylation in mdx mouse muscular dystrophy Muscle Nerve 1988 11 852 856 10.1002/mus.880110809 3173410

[B32] Salimena MC Lagrota-Candido J Quirico-Santos T Gender dimorphism influences extracellular matrix expression and regeneration of muscular tissue in mdx dystrophic mice Histochem Cell Biol 2004 122 435 444 15452719 10.1007/s00418-004-0707-8

[B33] Cros D Harnden P Pellissier JF Serratrice G Muscle hypertrophy in Duchenne muscular dystrophy. A pathological and morphometric study J Neurol 1989 236 43 47 10.1007/BF00314217 2915226

[B34] Murphy MM Lawson JA Mathew SJ Hutcheson DA Kardon G Satellite cells, connective tissue fibroblasts and their interactions are crucial for muscle regeneration Development 2011 138 3625 3637 10.1242/dev.064162 21828091 PMC3152921

[B35] Mann CJ Perdiguero E Kharraz Y Aguilar S Pessina P Serrano AL Munoz-Canoves P Aberrant repair and fibrosis development in skeletal muscle Skelet Muscle 2011 1 21 10.1186/2044-5040-1-21 21798099 PMC3156644

[B36] Hamer PW McGeachie JM Davies MJ Grounds MD Evans Blue Dye as an in vivo marker of myofibre damage: optimising parameters for detecting initial myofibre membrane permeability J Anat 2002 200 69 79 10.1046/j.0021-8782.2001.00008.x 11837252 PMC1570883

[B37] Peter AK Crosbie RH Hypertrophic response of Duchenne and limb-girdle muscular dystrophies is associated with activation of Akt pathway Exp Cell Res 2006 312 2580 2591 10.1016/j.yexcr.2006.04.024 16797529

[B38] Halevy O Piestun Y Allouh MZ Rosser BW Rinkevich Y Reshef R Rozenboim I Wleklinski-Lee M Yablonka-Reuveni Z Pattern of Pax7 expression during myogenesis in the posthatch chicken establishes a model for satellite cell differentiation and renewal Dev Dyn 2004 231 489 502 10.1002/dvdy.20151 15390217

[B39] Reimann J Irintchev A Wernig A Regenerative capacity and the number of satellite cells in soleus muscles of normal and mdx mice Neuromuscul Disord 2000 10 276 282 10.1016/S0960-8966(99)00118-2 10838255

[B40] Seale P Sabourin LA Girgis-Gabardo A Mansouri A Gruss P Rudnicki MA Pax7 is required for the specification of myogenic satellite cells Cell 2000 102 777 786 10.1016/S0092-8674(00)00066-0 11030621

[B41] Heo K Park KA Kim YH Kim SH Oh YS Kim IH Ryu SH Suh PG Sphingosine 1-phosphate induces vascular endothelial growth factor expression in endothelial cells BMB Rep 2009 42 685 690 10.5483/BMBRep.2009.42.10.685 19874715

[B42] Kimura T Watanabe T Sato K Kon J Tomura H Tamama K Kuwabara A Kanda T Kobayashi I Ohta H Ui M Okajima F Sphingosine 1-phosphate stimulates proliferation and migration of human endothelial cells possibly through the lipid receptors, Edg-1 and Edg-3 Biochem J 2000 348 Pt 1 71 76 10794715 PMC1221037

[B43] Rikitake Y Hirata K Kawashima S Ozaki M Takahashi T Ogawa W Inoue N Yokoyama M Involvement of endothelial nitric oxide in sphingosine-1-phosphate-induced angiogenesis Arterioscler Thromb Vasc Biol 2002 22 108 114 10.1161/hq0102.101843 11788469

[B44] Yeh HI Dupont E Coppen S Rothery S Severs NJ Gap junction localization and connexin expression in cytochemically identified endothelial cells of arterial tissue J Histochem Cytochem 1997 45 539 550 10.1177/002215549704500406 9111232

[B45] Beenakker EA Maurits NM Fock JM Brouwer OF van der Hoeven JH Functional ability and muscle force in healthy children and ambulant Duchenne muscular dystrophy patients Eur J Paediatr Neurol 2005 9 387 393 10.1016/j.ejpn.2005.06.004 16102988

[B46] Lynch GS Hinkle RT Chamberlain JS Brooks SV Faulkner JA Force and power output of fast and slow skeletal muscles from mdx mice 6–28 months old J Physiol 2001 535 591 600 10.1111/j.1469-7793.2001.00591.x 11533147 PMC2278782

[B47] Nagata Y Partridge TA Matsuda R Zammit PS Entry of muscle satellite cells into the cell cycle requires sphingolipid signaling J Cell Biol 2006 174 245 253 10.1083/jcb.200605028 16847102 PMC2064184

[B48] Frank SJ Engel I Rutledge TM Letourneur F Structure/function analysis of the invariant subunits of the T cell antigen receptor Semin Immunol 1991 3 299 311 1724736

[B49] Tajbakhsh S Bober E Babinet C Pournin S Arnold H Buckingham M Gene targeting the myf-5 locus with nlacZ reveals expression of this myogenic factor in mature skeletal muscle fibres as well as early embryonic muscle Dev Dyn 1996 206 291 300 10.1002/(SICI)1097-0177(199607)206:3<291::AID-AJA6>3.0.CO;2-D 8896984

[B50] Beauchamp JR Heslop L Yu DS Tajbakhsh S Kelly RG Wernig A Buckingham ME Partridge TA Zammit PS Expression of CD34 and Myf5 defines the majority of quiescent adult skeletal muscle satellite cells J Cell Biol 2000 151 1221 1234 10.1083/jcb.151.6.1221 11121437 PMC2190588

[B51] Day K Shefer G Richardson JB Enikolopov G Yablonka-Reuveni Z Nestin-GFP reporter expression defines the quiescent state of skeletal muscle satellite cells Dev Biol 2007 304 246 259 10.1016/j.ydbio.2006.12.026 17239845 PMC1888564

[B52] Rosen H Gonzalez-Cabrera PJ Sanna MG Brown S Sphingosine 1-phosphate receptor signaling Annu Rev Biochem 2009 78 743 768 10.1146/annurev.biochem.78.072407.103733 19231986

[B53] Liu CH Thangada S Lee MJ Van Brocklyn JR Spiegel S Hla T Ligand-induced trafficking of the sphingosine-1-phosphate receptor EDG-1 Mol Biol Cell 1999 10 1179 1190 10.1091/mbc.10.4.1179 10198065 PMC25247

[B54] Oo ML Chang SH Thangada S Wu MT Rezaul K Blaho V Hwang SI Han DK Hla T Engagement of S1P(1)-degradative mechanisms leads to vascular leak in mice J Clin Invest 2011 121 2290 2300 10.1172/JCI45403 21555855 PMC3104755

[B55] Estrada R Wang L Jala VR Lee JF Lin CY Gray RD Haribabu B Lee MJ Ligand-induced nuclear translocation of S1P(1) receptors mediates Cyr61 and CTGF transcription in endothelial cells Histochem Cell Biol 2009 131 239 249 10.1007/s00418-008-0521-9 18936953 PMC2861785

[B56] Kluk MJ Hla T Role of the sphingosine 1-phosphate receptor EDG-1 in vascular smooth muscle cell proliferation and migration Circ Res 2001 89 496 502 10.1161/hh1801.096338 11557736

[B57] Robert P Tsui P Laville MP Livi GP Sarau HM Bril A Berrebi-Bertrand I EDG1 receptor stimulation leads to cardiac hypertrophy in rat neonatal myocytes J Mol Cell Cardiol 2001 33 1589 1606 10.1006/jmcc.2001.1433 11549339

[B58] Bodine SC Stitt TN Gonzalez M Kline WO Stover GL Bauerlein R Zlotchenko E Scrimgeour A Lawrence JC Glass DJ Yancopoulos GD Akt/mTOR pathway is a crucial regulator of skeletal muscle hypertrophy and can prevent muscle atrophy in vivo Nat Cell Biol 2001 3 1014 1019 10.1038/ncb1101-1014 11715023

[B59] Lai KM Gonzalez M Poueymirou WT Kline WO Na E Zlotchenko E Stitt TN Economides AN Yancopoulos GD Glass DJ Conditional activation of akt in adult skeletal muscle induces rapid hypertrophy Mol Cell Biol 2004 24 9295 9304 10.1128/MCB.24.21.9295-9304.2004 15485899 PMC522257

[B60] Thomas G The S6 kinase signaling pathway in the control of development and growth Biol Res 2002 35 305 313 12415748 10.4067/s0716-97602002000200022

[B61] Anderson LV Davison K Moss JA Young C Cullen MJ Walsh J Johnson MA Bashir R Britton S Keers S Argov Z Mahjneh I Fougerousse F Beckmann JS Bushby KM Dysferlin is a plasma membrane protein and is expressed early in human development Hum Mol Genet 1999 8 855 861 10.1093/hmg/8.5.855 10196375

[B62] Ho M Post CM Donahue LR Lidov HG Bronson RT Goolsby H Watkins SC Cox GA Brown RH Jr Disruption of muscle membrane and phenotype divergence in two novel mouse models of dysferlin deficiency Hum Mol Genet 2004 13 1999 2010 10.1093/hmg/ddh212 15254015

[B63] Millay DP Maillet M Roche JA Sargent MA McNally EM Bloch RJ Molkentin JD Genetic manipulation of dysferlin expression in skeletal muscle: novel insights into muscular dystrophy Am J Pathol 2009 175 1817 1823 10.2353/ajpath.2009.090107 19834057 PMC2774048

[B64] Han R Rader EP Levy JR Bansal D Campbell KP Dystrophin deficiency exacerbates skeletal muscle pathology in dysferlin-null mice Skelet Muscle 2011 1 35 10.1186/2044-5040-1-35 22132688 PMC3287108

[B65] Aoki M Takahashi T [Mutational and clinical features of Japanese patients with dysferlinopathy (Miyoshi myopathy and limb girdle muscular dystrophy type 2B)] Rinsho shinkeigaku 2005 45 938 942 Article in Japanese 16447768

[B66] Fanin M Angelini C Muscle pathology in dysferlin deficiency Neuropathol Appl Neurobiol 2002 28 461 470 10.1046/j.1365-2990.2002.00417.x 12445162

[B67] Farini A Sitzia C Navarro C D’Antona G Belicchi M Parolini D Del Fraro G Razini P Bottinelli R Meregalli M Torrente Y Absence of T and B lymphocytes modulates dystrophic features in dysferlin deficient animal model Exp Cell Res 2012 318 1160 1174 10.1016/j.yexcr.2012.03.010 22465227

[B68] Mokhtarian A Lefaucheur JP Even PC Sebille A Hindlimb immobilization applied to 21-day-old mdx mice prevents the occurrence of muscle degeneration J Appl Physiol 1999 86 924 931 10066706 10.1152/jappl.1999.86.3.924

[B69] Torres LF Duchen LW The mutant mdx: inherited myopathy in the mouse. Morphological studies of nerves, muscles and end-plates Brain 1987 110 Pt 2 269 299 3567525 10.1093/brain/110.2.269

[B70] DiMario JX Uzman A Strohman RC Fiber regeneration is not persistent in dystrophic (MDX) mouse skeletal muscle Dev Biol 1991 148 314 321 10.1016/0012-1606(91)90340-9 1936568

[B71] Ikeda M Kihara A Igarashi Y Sphingosine-1-phosphate lyase SPL is an endoplasmic reticulum-resident, integral membrane protein with the pyridoxal 5′-phosphate binding domain exposed to the cytosol Biochem Biophys Res Commun 2004 325 338 343 10.1016/j.bbrc.2004.10.036 15522238

[B72] Hait NC Allegood J Maceyka M Strub GM Harikumar KB Singh SK Luo C Marmorstein R Kordula T Milstien S Spiegel S Regulation of histone acetylation in the nucleus by sphingosine-1-phosphate Science 2009 325 1254 1257 10.1126/science.1176709 19729656 PMC2850596

[B73] Colussi C Mozzetta C Gurtner A Illi B Rosati J Straino S Ragone G Pescatori M Zaccagnini G Antonini A Minetti G Martelli F Piaggio G Gallinari P Steinkuhler C Clementi E Dell'Aversana C Altucci L Mai A Capogrossi MC Puri PL Gaetano C HDAC2 blockade by nitric oxide and histone deacetylase inhibitors reveals a common target in Duchenne muscular dystrophy treatment Proc Natl Acad Sci USA 2008 105 19183 19187 10.1073/pnas.0805514105 19047631 PMC2614736

[B74] Iezzi S Di Padova M Serra C Caretti G Simone C Maklan E Minetti G Zhao P Hoffman EP Puri PL Sartorelli V Deacetylase inhibitors increase muscle cell size by promoting myoblast recruitment and fusion through induction of follistatin Dev Cell 2004 6 673 684 10.1016/S1534-5807(04)00107-8 15130492

[B75] Morissette MR Cook SA Buranasombati C Rosenberg MA Rosenzweig A Myostatin inhibits IGF-I-induced myotube hypertrophy through Akt Am J Physiol 2009 297 C1124 C1132 10.1152/ajpcell.00043.2009 PMC2777401 19759331

[B76] Zammit PS Partridge TA Sizing up muscular dystrophy Nat Med 2002 8 1355 1356 10.1038/nm1202-1355 12457175

[B77] Smyth SS Sciorra VA Sigal YJ Pamuklar Z Wang Z Xu Y Prestwich GD Morris AJ Lipid phosphate phosphatases regulate lysophosphatidic acid production and signaling in platelets: studies using chemical inhibitors of lipid phosphate phosphatase activity J Biol Chem 2003 278 43214 43223 10.1074/jbc.M306709200 12909631

[B78] Brinkmann V Billich A Baumruker T Heining P Schmouder R Francis G Aradhye S Burtin P Fingolimod (FTY720): discovery and development of an oral drug to treat multiple sclerosis Nat Rev Drug Discov 2010 9 883 897 10.1038/nrd3248 21031003

[B79] Kennedy PC Zhu R Huang T Tomsig JL Mathews TP David M Peyruchaud O Macdonald TL Lynch KR Characterization of a sphingosine 1-phosphate receptor antagonist prodrug J Pharmacol Exp Ther 2011 338 879 889 10.1124/jpet.111.181552 21632869 PMC3164350

