# Supplementary material for: Increased sphingosine-1-phosphate improves muscle regeneration in acutely injured mdx mice
Source: Skelet Muscle. 2013 Aug 1;3:20. doi: 10.1186/2044-5040-3-20 (PMC3750760; doi:10.1186/2044-5040-3-20)
Supplement: Additional file 1: Figure S1 — Treatment with THI lowers mdx plasma platelet levels. Figure S2. THI alters the expression of S1P regulatory gene in mdx muscle. Figure S3. THI does not alter S1P plasma levels but lowers plasma CK activity. Figure S4. Muscle weight is preserved and hydroxy proline is reduced in injured muscles from THI treated mdx mice. Figure S5. Fibrosis is lower in uninjured mdx TA muscles with THI treatment. Figure S6. The number of centrally nucleated muscle fibers does not change with THI. Figure S7. Diaphragm muscle fibers size increases with THI treatment. Figure S8. THI treated mdx mice have an elevated number of Pax7+ satellite cells. Figure S9. The microvasculature of mdx muscles did not increase with THI. Figure S10. CTX injected in the TA reaches and also damages the EDL. Figure S11. THI treatment did not reduce T-cells in mdx diaphragms. Figure S12. Montages covering entire cross-sectional areas of each TA from S1P and vehicle treated mdx4CV:Myf5nlacZ/+ animals, were created by combining individual 10x photos. Figure S13. (A) Quantification of centrally nucleated muscle fibers from the same injured TAs presented in Figure 5, coincides with the number of newly regenerated fibers (eMyHC+ fibers) observed in S1P injected TA muscles. (B) Quantification of the minimum diameter of the largest eMyHC+ myofibers represented in Figure 5, indicates a significant increase in regenerated fiber size with S1P treatment. Figure S14. The expression of S1P receptors is reduced in mdx muscle cells. Figure S15. Direct S1P administration results in elevated levels of phosphorylation S1PR1 in mdx muscles. Figure S16. S1P promotes muscle regeneration in the A/J mouse model of dysferlinopathy. Table S1. Average number of Evans Blue+ muscle fibers within each muscle group. [file 2044-5040-3-20-S1.pdf]

Figure S1. Treatment with THI lowers *mdx* plasma platelet levels. Measurements from peripheral blood indicates *mdx*^{4cv} (n=3, 1.5 MO)mice have a significantly elevated number of platelets. This this number is reduced to near wt (n=4) levels 12 hours post THI treatment; 2x 250µl IP injections of 0.15mg/ml THI, 6 hours apart. Note the analysis was done 12 hours following the second THI injection. By ANOVA analysis, there was a significant difference (P<0.005) between pre/post treatment and wt. * denotes P<0.05. Error bars represent SEM.

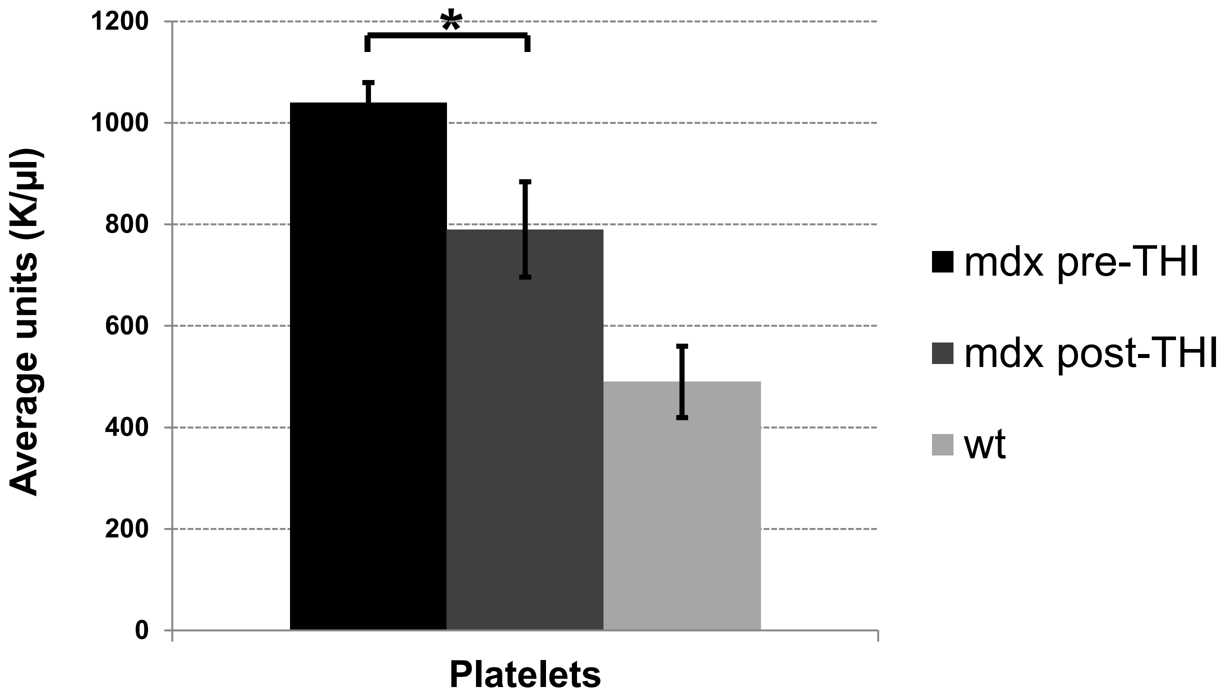

Figure S2. THI alters the expression of S1P regulatory gene in *mdx* muscle. Quantitative reverse transcription-PCR analysis of TA muscles from 3.5 MO mdx^{4cv} male mice (n=6) mice, injured (Left) vs. uninjured (Right) and treated with THI or vehicle (PBS) for 3 days post CTX injection. Muscles were harvested at day 4 post CTX injury for analysis of (*A*) *S1P phosphatase 1* and *lyase,* and (*B*) *S1P kinase 1* and *2.* * denotes P<0.05, ** P<0.005, and *** P<0.0005. Expression was normalized to ribosomal subunit *18S* expression. Error bars represent SEM.

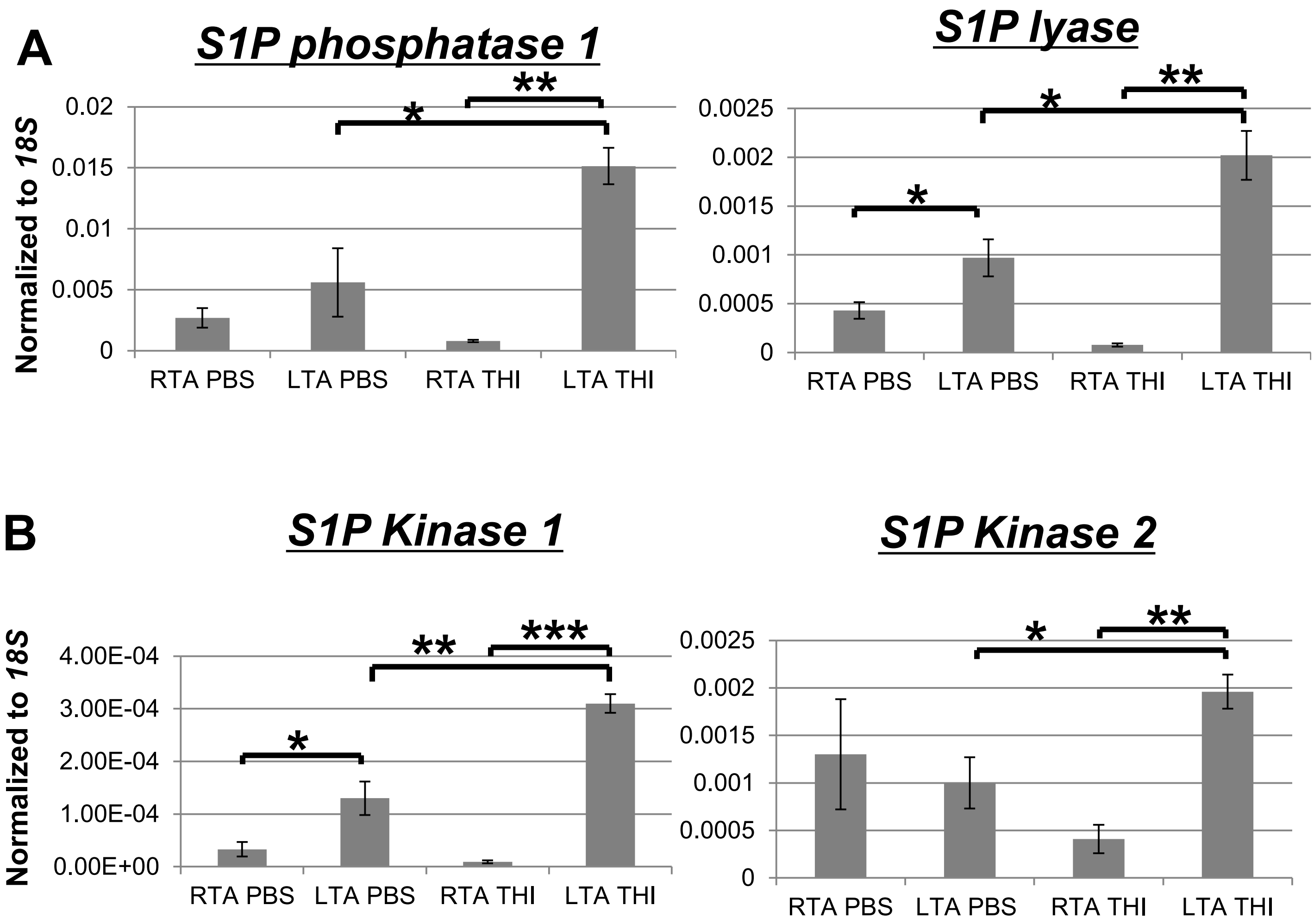

Figure S3. THI does not alter S1P plasma levels but lowers plasma CK activity. S1P levels and creatine kinase (CK) activity were measured from 5 MO *mdx*[4cv] mice (n=6) mice injured and treated with THI or vehicle (PBS) for 3 days post CTX injection. Plasma was collected and analyzed on day 4 post injury. (*A*) LC-MS/MS analysis indicates S1P plasma levels do not increase with THI treatment. (*B*) Baseline creatine kinase activity (Left graph) and normalized to body weight (Right graph), was lower but not significantly different with the aforementioned THI treatment regiment following CTX injury. The weight of these animals just prior to the blood draw (represented in table) was also not significantly different with THI treatment. Error bars represent SEM.

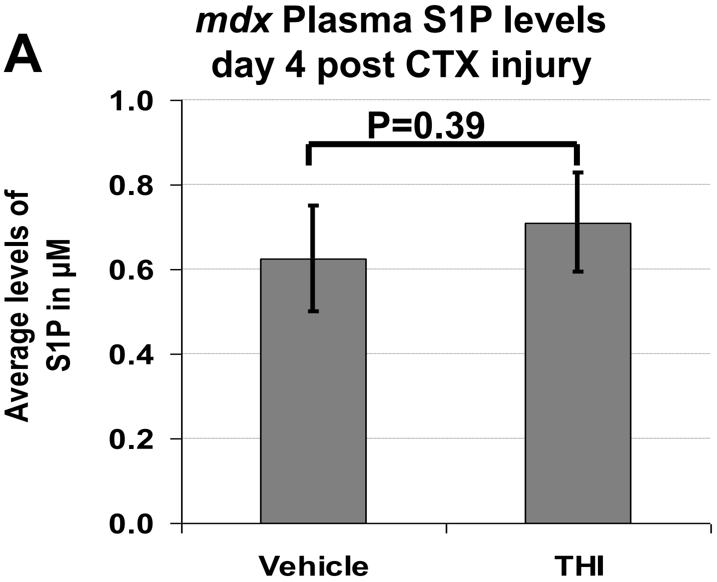

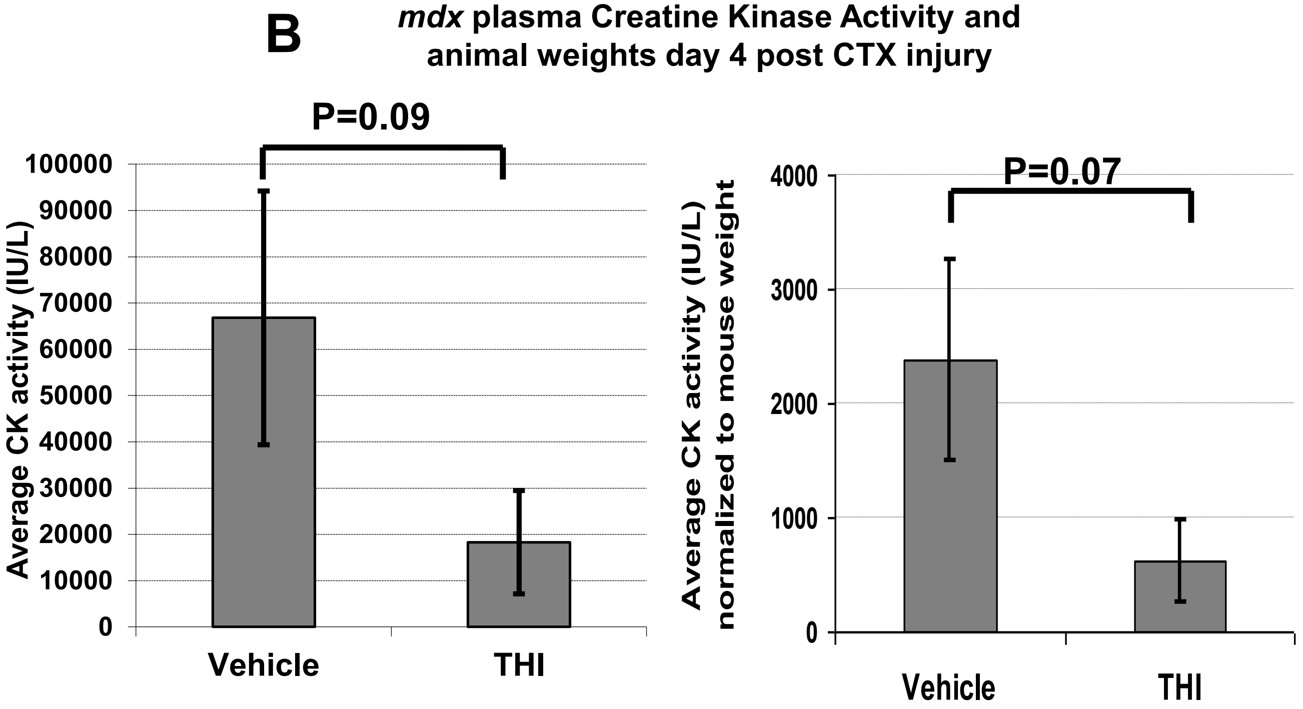

	Vehicle	THI
**Average animal weight**	27.3 (±1.2 SEM)	28.7 (±1.9 SEM)

Figure S4. Muscle weight is preserved and hydroxy proline is reduced in injured muscles from THI treated *mdx* mice.
(*A*) The ratio of dry weight from each CTX injured over uninjured muscles (TAs and quadriceps) normalized to body weight of 16 MO *mdx*^{4cv} males. Although individual muscle weights were not significantly different, the ratio between injured/uninjured muscles was significantly lower in vehicle vs. THI treated quadriceps. (*B*) Hydroxy proline analysis of CTX TA's from 16 MO *mdx*^{4cv} animals. This analysis was done blinded by AAA service laboratory (Damascus, OR). Error bars represent SEM.

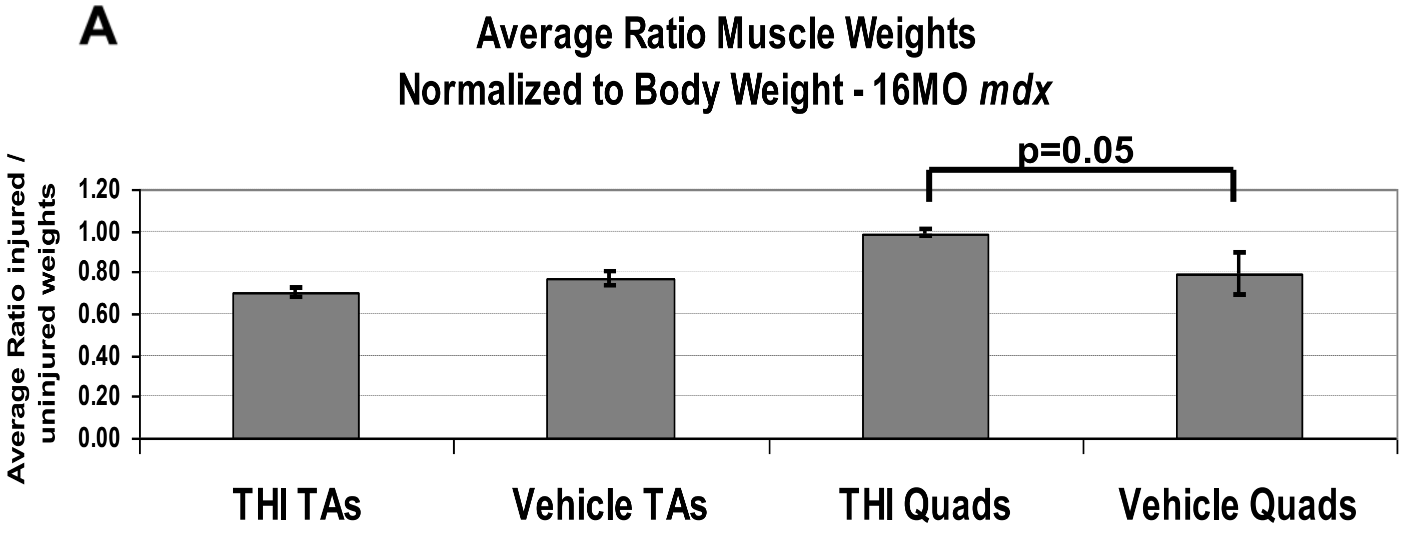

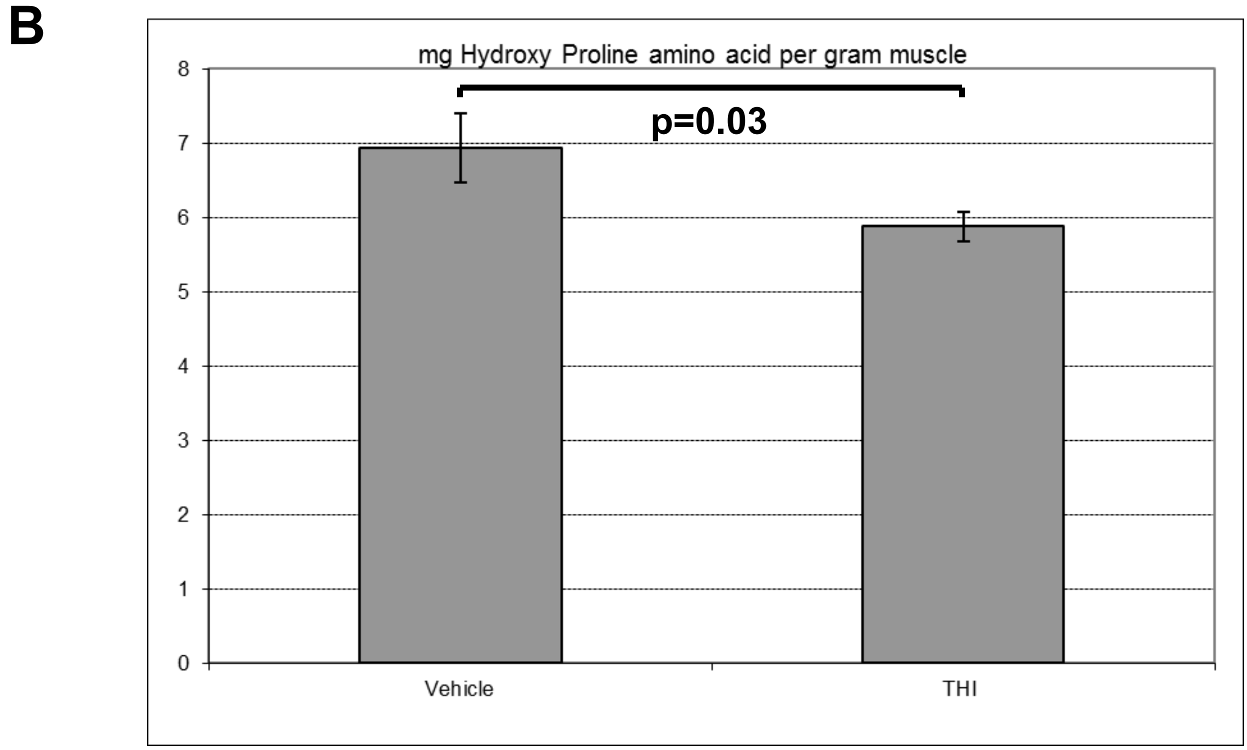

Figure S5. Fibrosis is lower in uninjured *mdx* TA muscles with THI treatment. Representative staining of fast green and picrosirius red, highlighting lower fibrosis in the uninjured (right limb) TAs of 11 MO female mice, quantified in Figure 2B. Note, the intensity of the red staining is indicative of collagen density. Scale bars=50μm.

THI uninjured TA 11 MO ♀

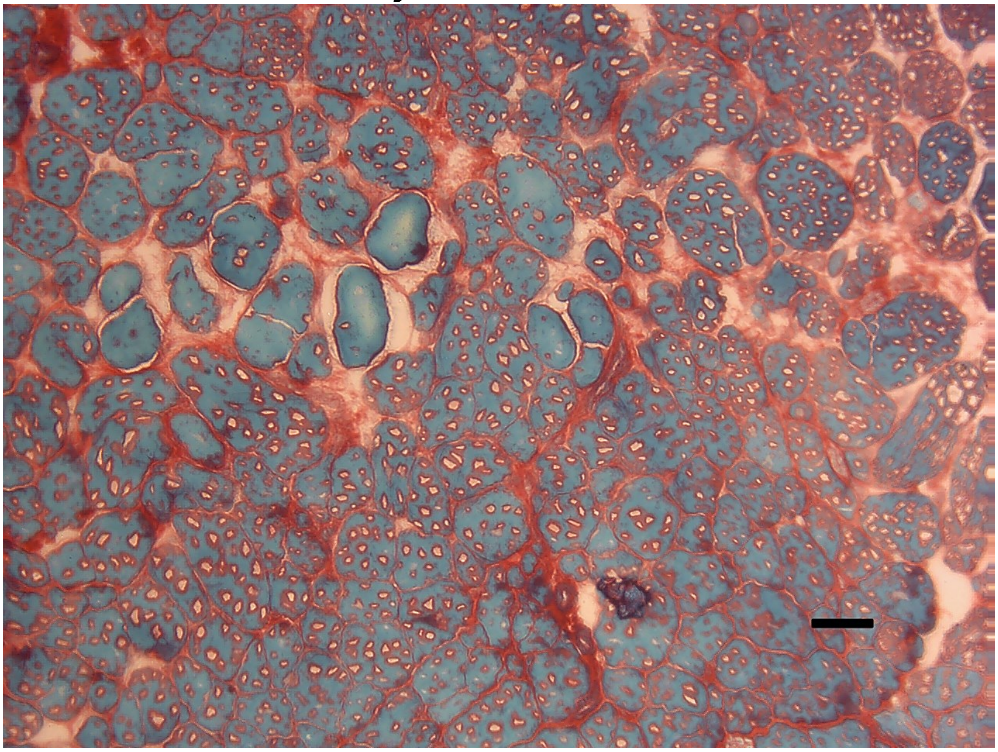

Vehicle uninjured TA 11 MO ♀

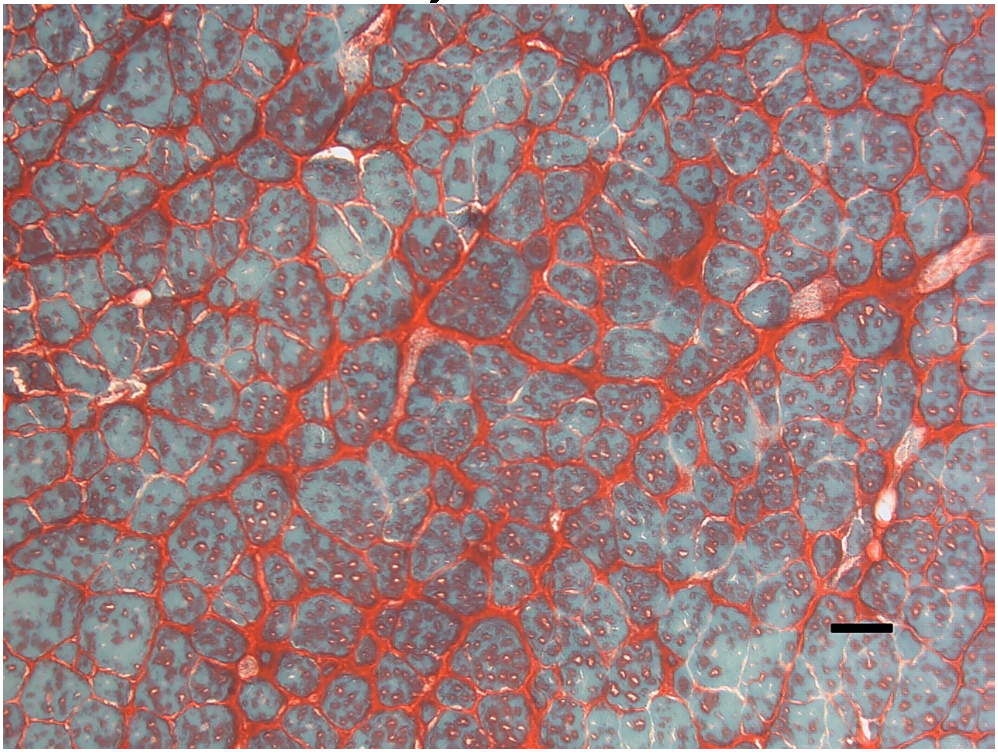

Figure S6. The number of centrally nucleated muscle fibers does not change with THI.
THI treatment did not change the proportion of muscle fibers with central nuclei. The total number of myofibers and the number of myofibers that were centrally nucleated were counted on the cross-sections of uninjured (*A*) and CTX injured (*B*) quadriceps muscles, stained for laminin and DAPI, from *mdx^{4cv}* mice (11 MO females, n=3) treated with THI or vehicle (IP injection, 250ul of 0.15 mg/ml THI or PBS as vehicle, twice daily, 6 hours apart for 3 days and samples analyzed on day 18). A total of 816 (THI) and 833 (vehicle) myofibers in uninjured and 747 (THI) and 811 (vehicle) myofibers were counted in injured quadriceps.

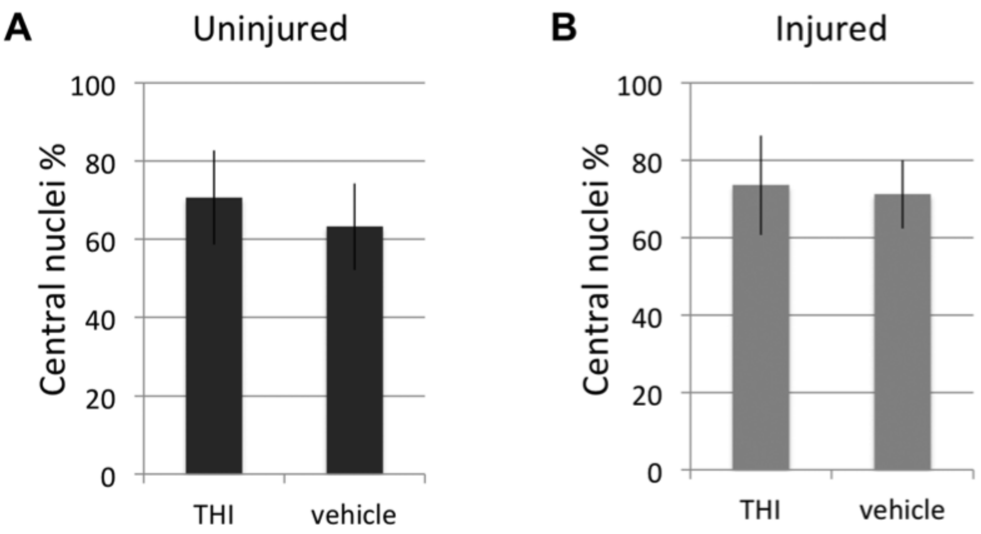

Figure S7. Diaphragm muscle fibers size increases with THI treatment.
Minimum muscle fiber diameter quantification of diaphragm muscles from THI and vehicle treated 11MO *mdx*^{4cv} females (n=6). As indicated by the distributions, mean and median values of muscle fiber minimum diameters, there is an overall increase in muscle fiber size with THI treatment..

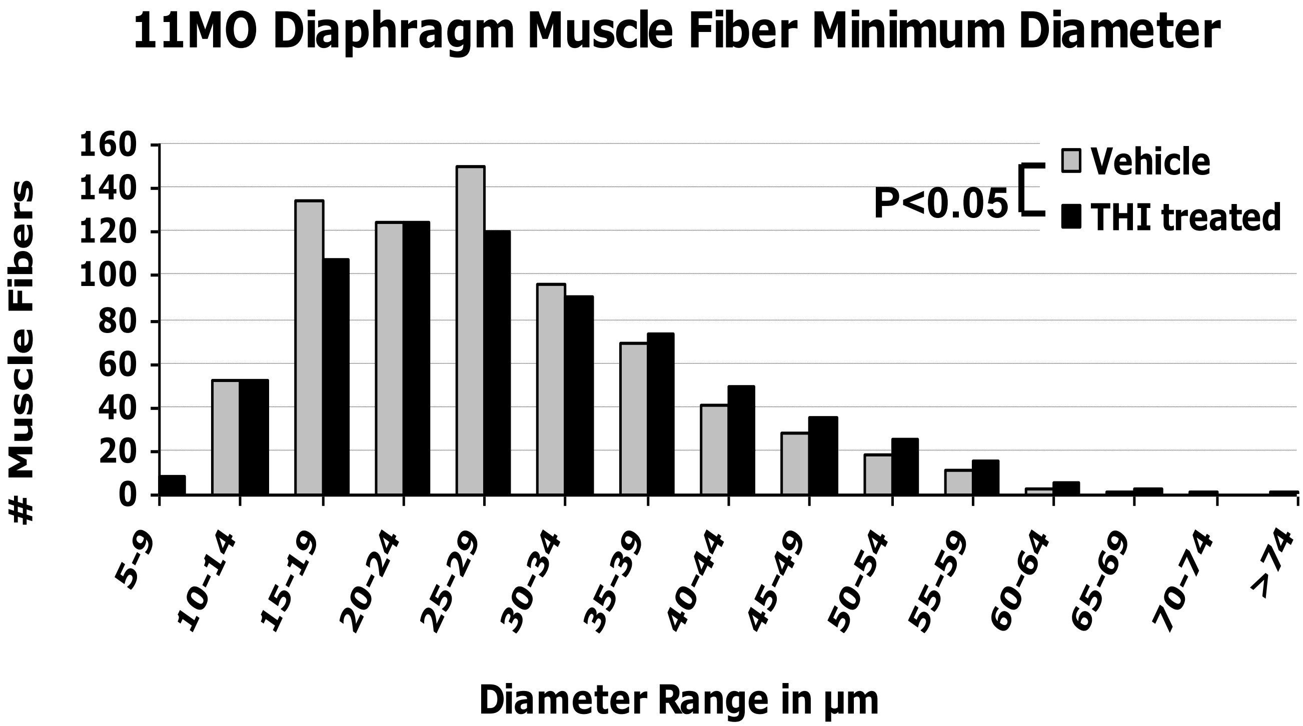

	Vehicle	THI
mean	27.4	28.6
median	25.8	26.4
Counts	737	713

Figure S8. THI treated *mdx* mice have an elevated number of Pax7+ satellite cells.
(*A*) Staining for Pax7 (AlexaFluor488 depicted in green) reveals a greater number of satellite cells, collectively in THI treated limb muscles of 11MO *mdx* mice. Representative nuclear Pax7 staining of CTX injured TA muscles from THI (left column) and Vehicle treated (right column) *mdx*^{4cv} animals. Arrowheads designate Pax7+ nuclei. Scale bar= 50µm. (*B*) Quantification of Pax7+ nuclei reveals almost a 2 fold increase in satellite cells, in TA and quadriceps muscles collectively. Error bars represent SEM

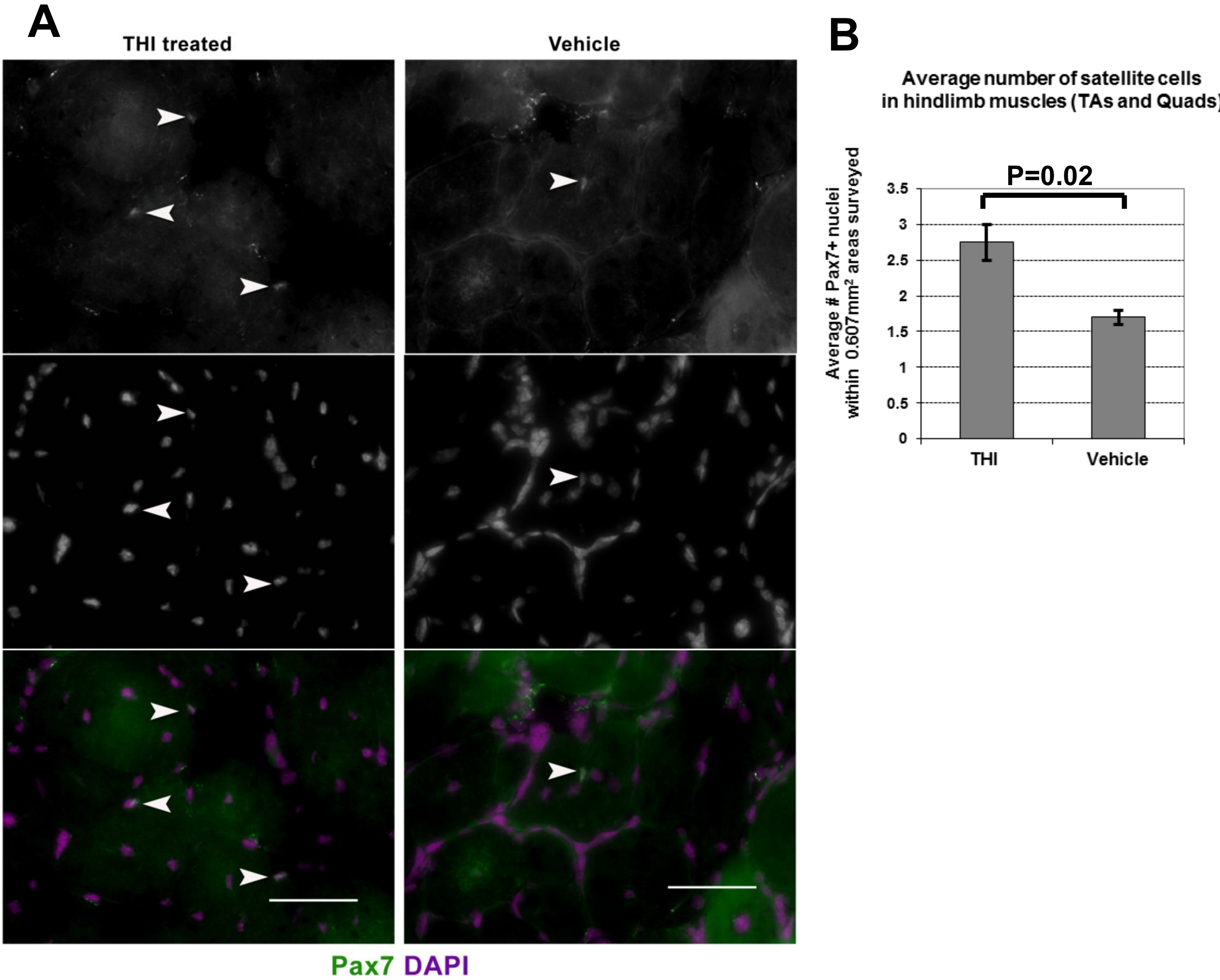

Figure S9. The microvasculature of *mdx* muscles did not increase with THI.
(*A*) Histological quantification of BS1+ capillaries indicates that THI treatment does not increase the muscle microvasculature in injured TA muscles from 11MO *mdx4cv* mice (n=6). Muscle cross-sections were stained with BS1 to identify microvessels and WGA as a counterstain for muscle fibers. Quantification of BS1+ microvessels was done using ImageJ and normalized to the number of muscle fibers per field. Scale bar= 50μm. (*B*) Quantitative RT-PCR analysis of *eNOS* and *CD31* in CTX injured (day 4 post injury) and uninjured contralateral TA muscles, suggests that the microvasculature does not expand with THI treatment. Expression was normalized to *GAPDH* expression. Error bars represent SEM.

A

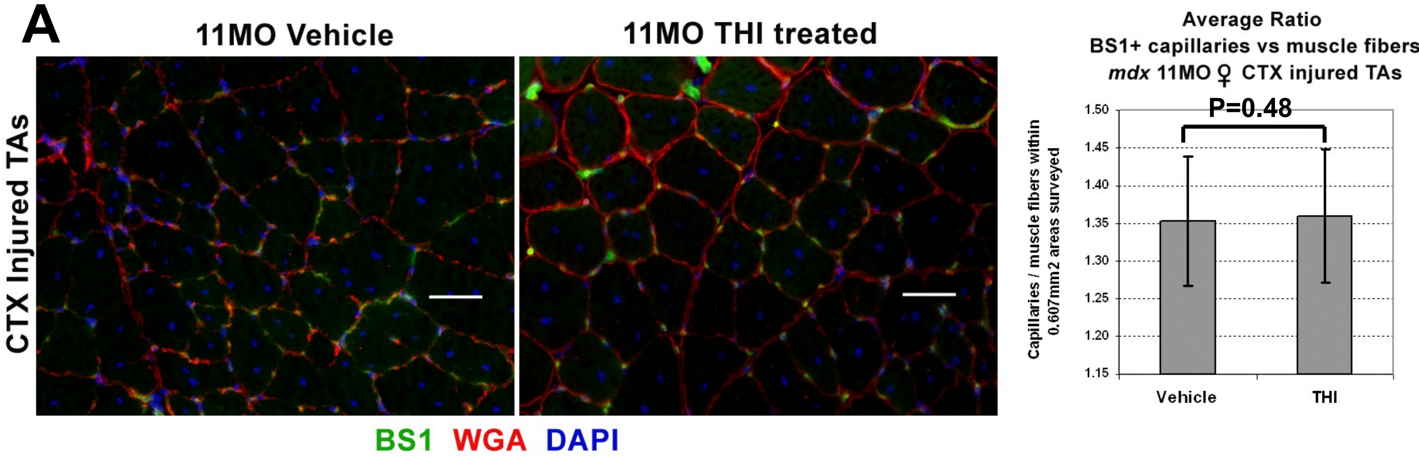

B

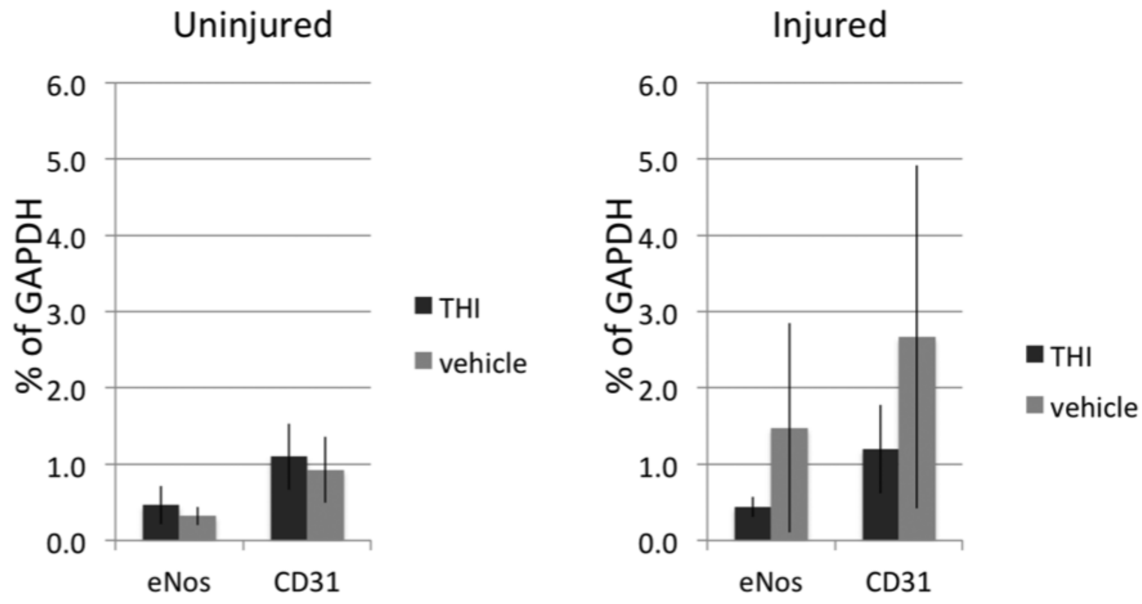

Figure S10. CTX injected in the TA reaches and also damages the EDL.
Assessment of EDL injury following CTX injection in TA muscles of *mdx* C57BL/k10 (n=4) mice indicates EDL muscles are damaged but not penetrated by the needle. (*A*) Photograph of the whole muscle shows TA and EDL muscles from uninjured contralateral and injured limb muscles respectively, as indicated by the presence of Evans Blue dye. (*B*) Histological analysis shows the presence of India ink (arrowheads) from the injection of CTX, present only in TA muscles. This indicates, that despite the resulting damage to both muscles, the needle used for intramuscular injection of CTX did not penetrate the EDL. Scale bar= 50µm.

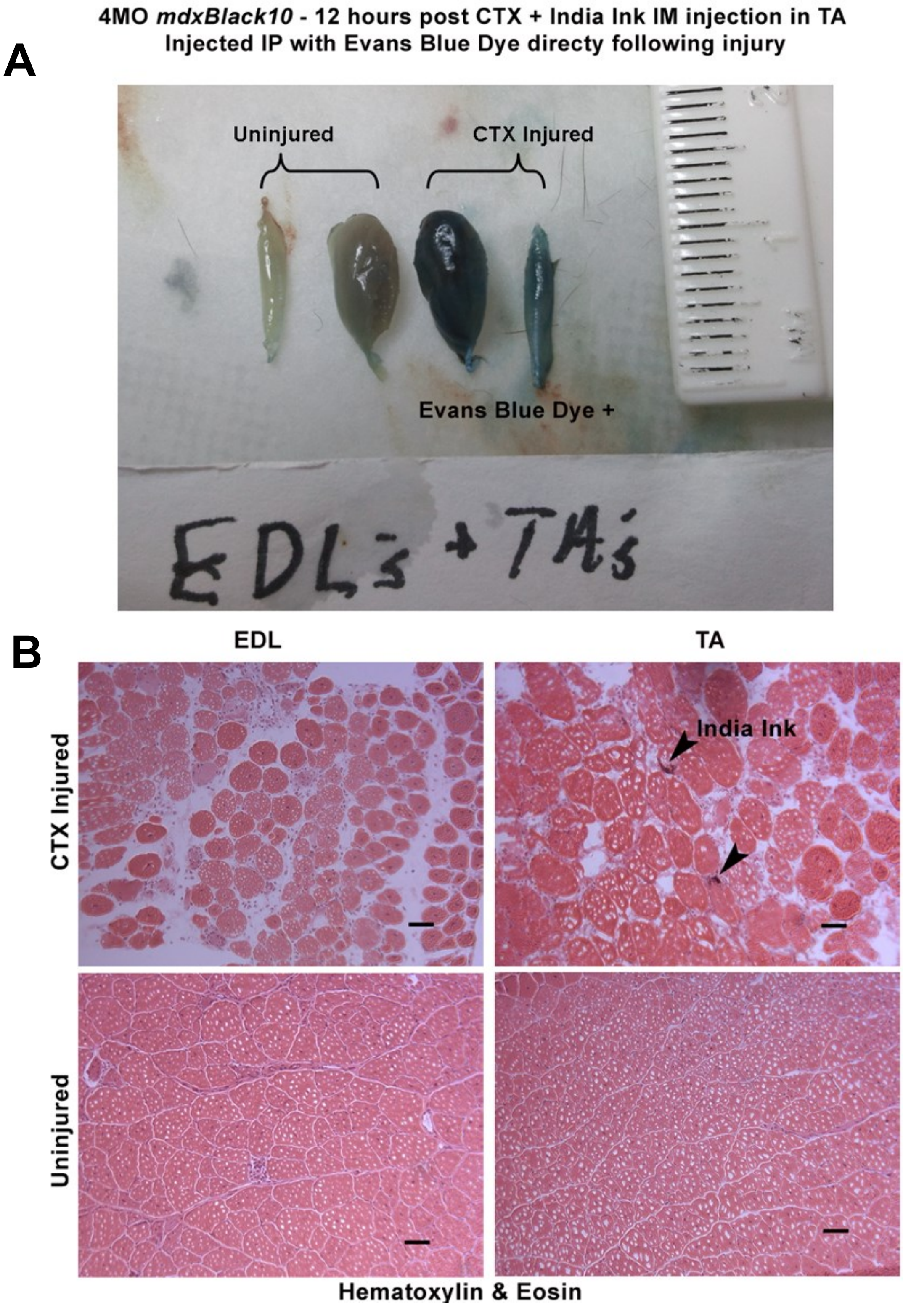

Figure S11. THI treatment did not reduce T-cells in *mdx* diaphragms.
Quantification of CD3e, a marker of T-cells, surveyed by histological staining in diaphragm muscles was similar between THI and vehicle treated *mdx* C57BL/k10 mice (n=8, 5 MO from Figure4B). Results indicate little difference between animals treated for 2 weeks via daily IP injections, with THI or vehicle. Error bars represent SEM.

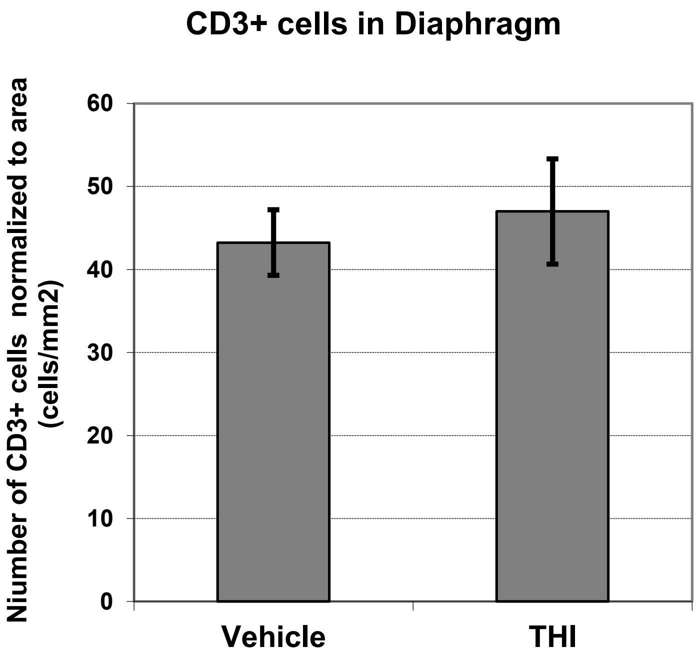

Figure S12. Montages covering entire cross-sectional areas of each TA from S1P and vehicle treated *mdx4CV:Myf5*$^{nlacZ/+}$ animals, were created by combining individual 10x photos. Pictures are representative of 8μm thick, x-gal stained sections. Individual β-gal+ nuclei were counted from montages using the ImageJ v1.40 cell counter plugin. Scale bar = 0.5mm.

Vehicle

3159 β-gal+ (*Myf5*$^{nlacZ/+}$) nuclei

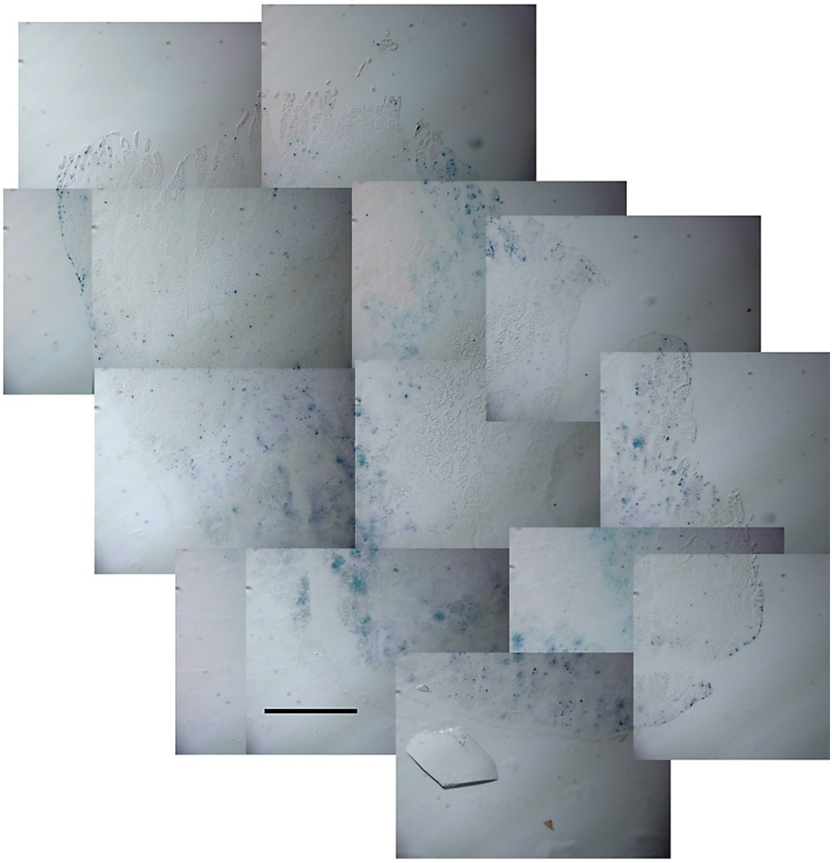

S1P injected

5338 β-gal+ (*Myf5*$^{nlacZ/+}$) nuclei

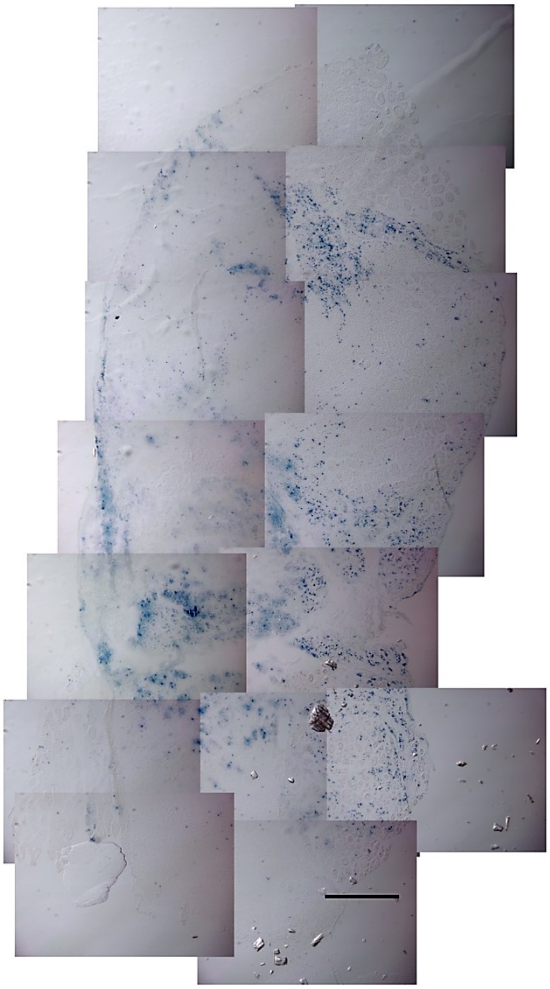

Figure S13. (*A*) Quantification of centrally nucleated muscle fibers from the same injured TAs presented in Figure 5, coincides with the number of newly regenerated fibers (eMyHC+ fibers) observed in S1P injected TA muscles. (*B*) Quantification of the minimum diameter of the largest eMyHC+ myofibers represented in Figure 5, indicates a significant increase in regenerated fiber size with S1P treatment. Error bars represent SEM.

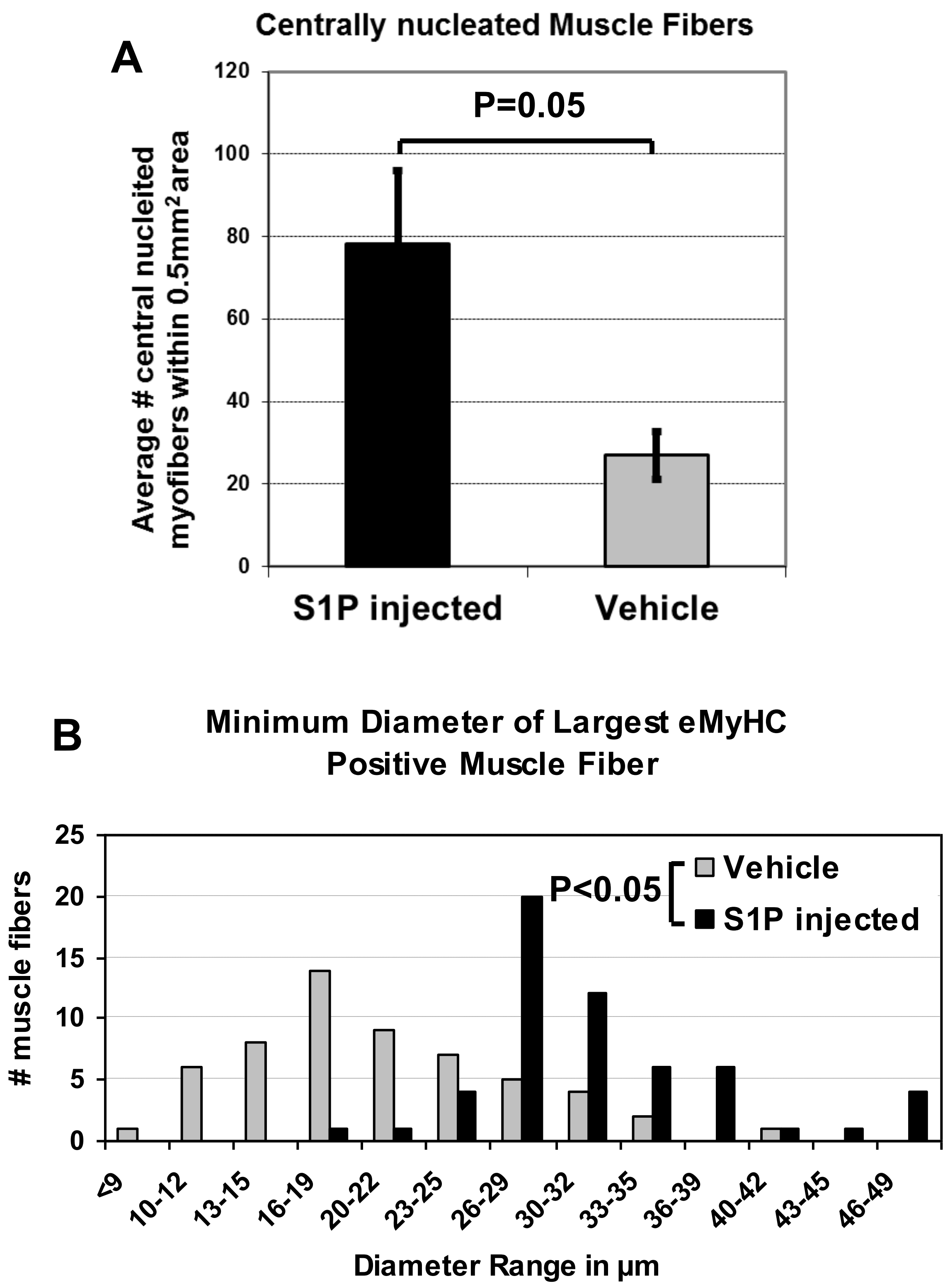

Figure S14. The expression of S1P receptors is reduced in *mdx* muscle cells. Quantitative RT-PCR of mononuclear cells digested from limb muscles (pooled TAs, Quadriceps, and Gastrocnemius muscles), reveals a significant reduction in expression of S1P receptors 1-3 (R1, R2, R3) in *mdx* (n=5) vs. wt (n=4) muscle cells.*, #, @ denote P<0.05 between *mdx* and wt for each respective receptor. Error bars represent SEM.

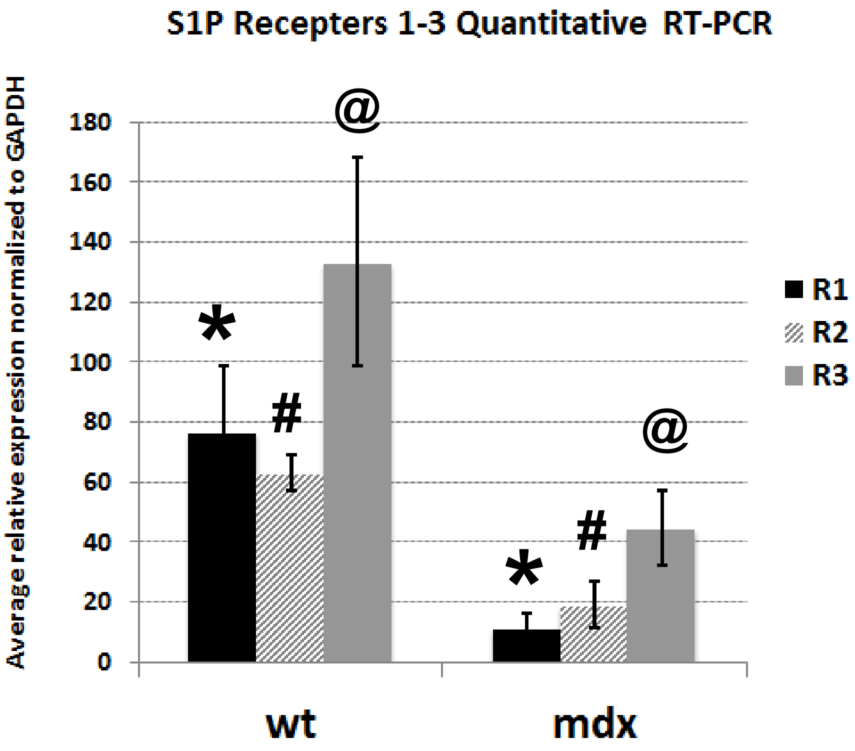

Figure S15. Direct S1P administration results in elevated levels of phosphorylation S1PR1 in *mdx* muscles.
Western blot analysis of S1P or vehicle injected TAs from *mdx*^{4cv} mice (n=3, 10 MO) for phosphorylated-S1PR1 indicated elevated levels with S1P treatment. Mice were injected in uninjured TAs with the same dose and regiment of S1P as depicted in Figure 5A, for 3 days and harvested on day 4 or analysis. Error bars represent SEM.

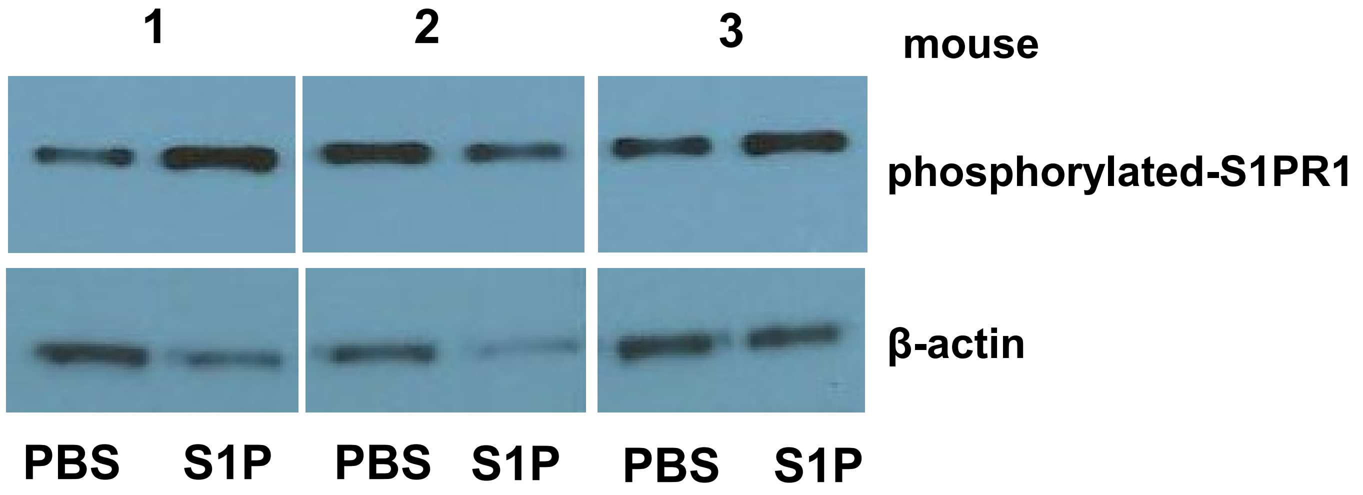

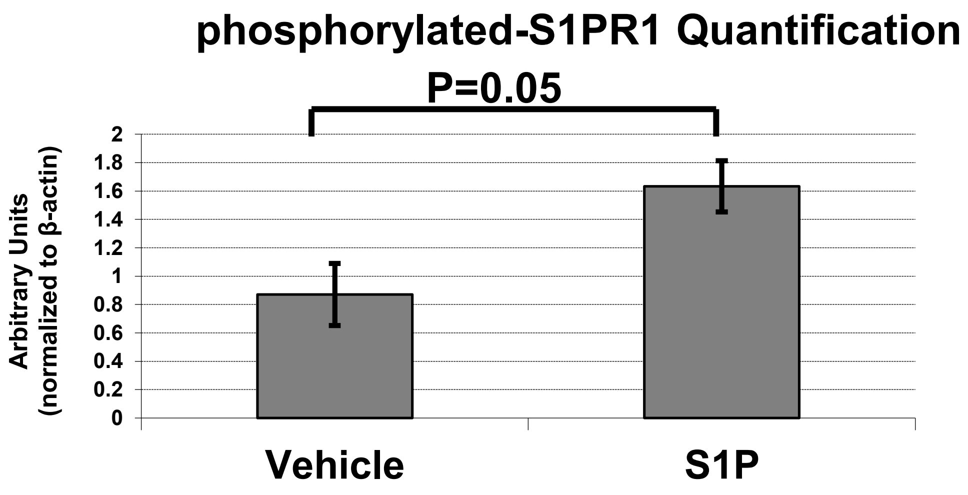

Figure S16. S1P promotes muscle regeneration in the A/J mouse model of dysferlinopathy. Following the same experimental design as figure 5A, both TAs from 9 MO A/J:SCID mice (n=4) were injected with CTX then subsequently injected with S1P (left TAs) or vehicle (right TAs) for the first three days following injury. In contrast to the previous experimental design which relied on the analysis of *Myf5*$^{nlacZ/+}$ reporter, TAs from AJ mice were analyzed at day 6 post injury in order to identify regenerating fibers and early accumulation of fibrosis. (*A*) Representative photograph of TAs from stained for picrosirius red and fast green. Scale bar = 50µm. (*B*) Fibrosis was quantified within in damaged/ regenerating regions of TA muscles. S1P injected TA muscles showed significantly less picrosirius red staining as compared to vehicle controls. (*C*) In conjunction with the decline in fibrosis, we also observed a significant increase of centrally nucleated muscle fibers indicating that S1P promoted muscle regeneration in acutely injured AJ muscles. Error bars represent SEM.

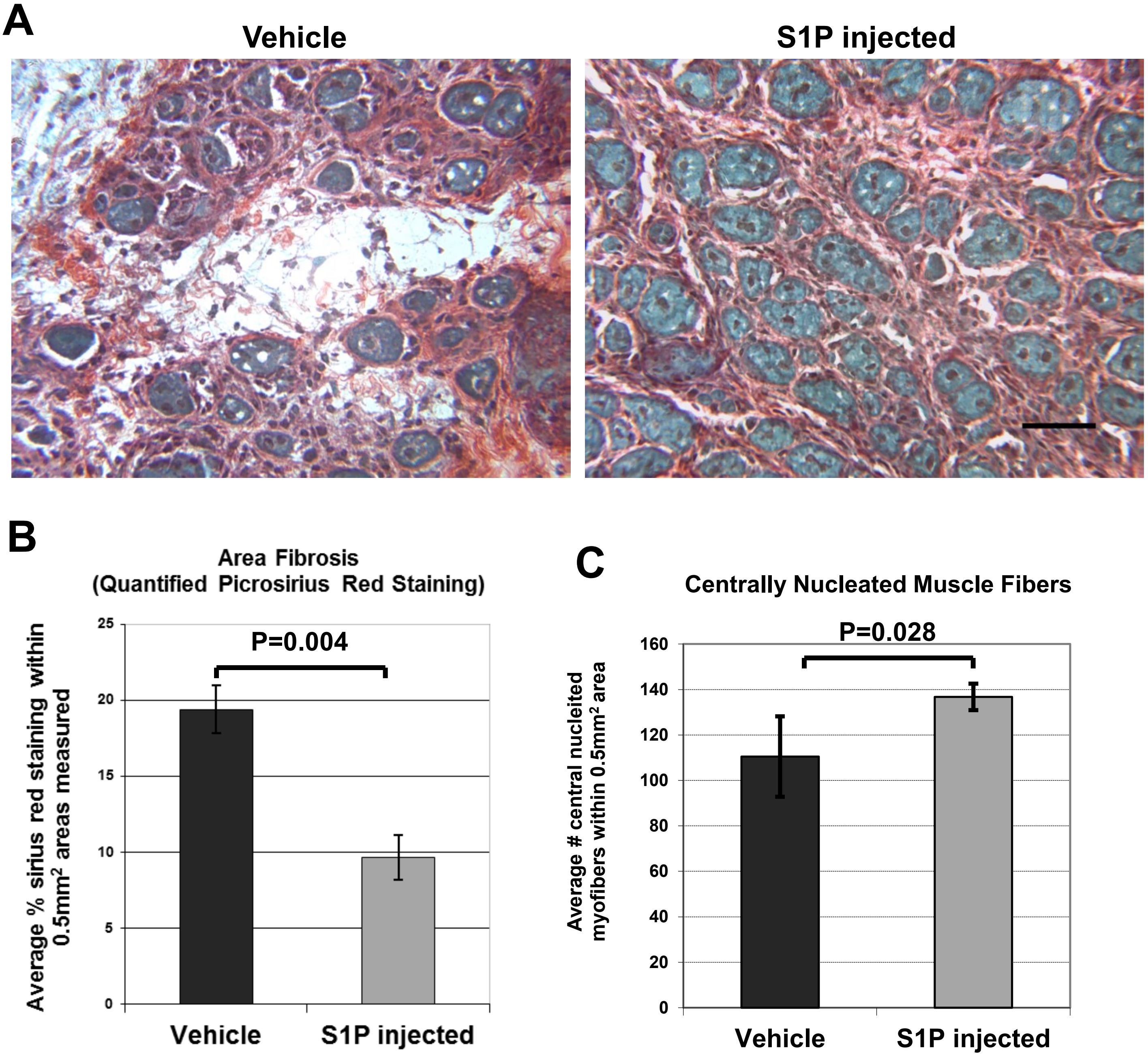

Table S1.
Average number of Evans Blue+ muscle fibers within each muscle group.
n=3 per condition

		**Injured Quads**	**Uninjured Quads**	**Injured TAs**	**Uninjured TAs**
**11MO ♀**	Vehicle	**45.0 (±16.3) ***	**9.8 (±1.0) ***	**19.3 (±5.7) ***	1.5 (±0.4)
	THI treated	**14 (±2.1) ***	**3.5 (±0.6) ***	**6.2(±2.4) ***	2.0 (±0.7)
**16 MO ♂**	Vehicle	161.5 (±43.3)	42.8 (±8.5)	18.5 (±4.2)	6.7 (±2.5)
	THI treated	248.7 (±67.3)	81.7 (±20.3)	38 (±15.3)	9 (±1.6)

The number of EBD+ fibers was quantified over the entire cross-section of each respective muscle.
*** P<0.05, ±SEM**
